# PPAD Activity Promotes Outer Membrane Vesicle Biogenesis and Surface Translocation by Porphyromonas gingivalis

**DOI:** 10.1128/JB.00343-20

**Published:** 2021-01-25

**Authors:** Danielle M. Vermilyea, M. Fata Moradali, Hey-Min Kim, Mary E. Davey

**Affiliations:** a Department of Oral Biology, College of Dentistry, University of Florida, Gainesville, Florida, USA; Brigham and Women's Hospital/Harvard Medical School

**Keywords:** *Bacteroidetes*, anaerobes, biofilms, biopearling, motility, proteases, protein secretion

## Abstract

Many bacteria switch between a sessile and a motile mode in response to environmental and host-related signals. Porphyromonas gingivalis, an oral anaerobe implicated in the etiology of chronic periodontal disease, has long been described as a nonmotile bacterium. And yet, recent studies have shown that under certain conditions, P. gingivalis is capable of surface translocation. Considering these findings, this work aimed to increase our understanding of how P. gingivalis transitions between sessile growth and surface migration. Here, we show that the peptidylarginine deiminase secreted by P. gingivalis (PPAD), an enzyme previously shown to be upregulated during surface translocation and to constrain biofilm formation, promotes surface translocation. In the absence of PPAD, the production of outer membrane vesicles (OMVs) was drastically reduced. In turn, there was a reduction in gingipain-mediated proteolysis and a reduced zone of hydration around the site of inoculation. Transcriptome sequencing (RNA-Seq) and metabolomics analyses also showed that these changes corresponded to a shift in arginine metabolism. Overall, this report provides new evidence for the functional relevance of PPAD and proteases, as well as the importance of PPAD activity in OMV biogenesis and release. Our findings support the model that citrullination is a critical mechanism during lifestyle transition between surface-attached growth and surface translocation by modulating OMV-mediated proteolysis and arginine metabolism.

**IMPORTANCE** Gram-negative bacteria produce nanosized OMVs that are actively released into their surroundings. The oral anaerobe P. gingivalis is prolific in OMV production, and many of the proteins packaged in these vesicles are proteolytic or protein-modifying enzymes. This includes key virulence determinants, such as the gingipains and PPAD (a unique peptidylarginine deiminase). Here, we show that PPAD activity (citrullination) is involved in OMV biogenesis. The study revealed an unusual mechanism that allows this bacterium to transform its surroundings. Since OMVs are detected in circulation and in systemic tissues, our study results also support the notion that PPAD activity may be a key factor in the correlation between periodontitis and systemic diseases, further supporting the idea of PPAD as an important therapeutic target.

## Abstract (precis)

Gram-negative bacteria produce nanosized OMVs that are actively released into their surroundings. The oral anaerobe P. gingivalis is prolific in OMV production, and many of the proteins packaged in these vesicles are proteolytic or protein-modifying enzymes.

## INTRODUCTION

The ability to transition between surface-attached growth and a motile state is an important adaptive mechanism for survival and persistence in some bacteria (1–3). Despite the fact that the periodontal pathogen Porphyromonas gingivalis has historically been described as nonmotile, given certain environmental parameters, this bacterium is capable of surface migration (4). Specifically, surface translocation has been demonstrated at the interface of soft agar and a glass or plastic surface. And yet, the underlying mechanisms involved in this lifestyle transition are largely unknown. In translocating cells, numerous genes are upregulated, including genes encoding a number of proteins secreted by the type IX secretion system (T9SS), a secretion system linked to gliding motility in other members of the phylum *Bacteroidetes* (5–8). The T9SS in P. gingivalis transports over 30 proteins that are delivered to the outer membrane (OM) and also released into the surrounding environment via outer membrane vesicles (OMVs) (9). T9SS cargo proteins include a variety of proteases, hemagglutinins, adhesins, and the peptidylarginine deiminase secreted by P. gingivalis (PPAD) (9). Importantly, it was previously noted that during the initial stage of surface translocation (hydration stage), *ppad* expression was upregulated 13-fold and the gingipains were upregulated up to 22-fold (4). Furthermore, formation of a hydration zone was characterized by hydrolysis and clearing of the red blood cells and it was proposed to be dependent on the cumulative activity of proteases and PPAD (4, 10). These findings are of particular interest given recent work showing that PPAD-mediated citrullination modulates biofilm development; specifically, deletion of *ppad* in strain 381 resulted in greater biofilm biomass due to the retention of gingipains and gingipain-derived adhesins within the matrix (11). Overall, the data support a model of cooperative function between gingipains and PPAD in modification of the surroundings and modulation of lifestyle transition.

Additionally, metabolomic analysis of translocating cells showed that arginine and putrescine were exhausted while citrulline accumulated in the extracellular milieu (4). The exhaustion of arginine and putrescine suggested that polyamine biosynthesis plays a role in surface translocation. Polyamines are polycationic molecules that are produced from amino acids. Spermidine has been reported to be the most prevalent polyamine in bacteria (12). For many species in the *Bacteroidetes* phylum, including P. gingivalis, spermidine is predicted to be synthesized from arginine via the carboxyspermidine dehydrogenase/carboxyspermidine decarboxylase (CASDH/CASDC) pathway, which includes putrescine as an intermediate (12, 13). The production of polyamines, specifically, spermidine, has been shown to modulate both biofilm development and motility in other bacteria (14–16). Though arginine and putrescine levels were exhausted from the surroundings by translocating P. gingivalis cells, spermidine levels were not reported. It is also known that PPAD can citrullinate both peptidylarginine and free arginine (17). Thus far, the effect of deleting *ppad* on peptidylarginine and/or arginine levels has not been investigated directly, and is it unclear what effect any resulting change in arginine concentration may have on polyamine biosynthesis.

Given that recent studies on PPAD in strain 381 have shown that biofilm formation is enhanced when *ppad* is deleted, that deletion of *ppad* inhibits the release of gingipains, and that the expression levels of PPAD and proteases are upregulated during surface translocation, our working hypothesis for this study was that PPAD-mediated citrullination and arginine metabolism play roles in modulating the transition between sessile growth and surface translocation. To test this hypothesis, surface translocation of fimbriated strain 381 and its corresponding *ppad* deletion mutant (381Δ*ppad*) was investigated using an anaerobic chamber slide system and time-lapse microscopy along with a combination of transcriptomic, genomic, and metabolomic analyses. Overall, our findings support the model that PPAD activity is involved in surface conditioning, modulation of arginine metabolism, and OMV biogenesis.

## RESULTS

### PPAD facilitates P. gingivalis surface translocation.

**FIG 1:**
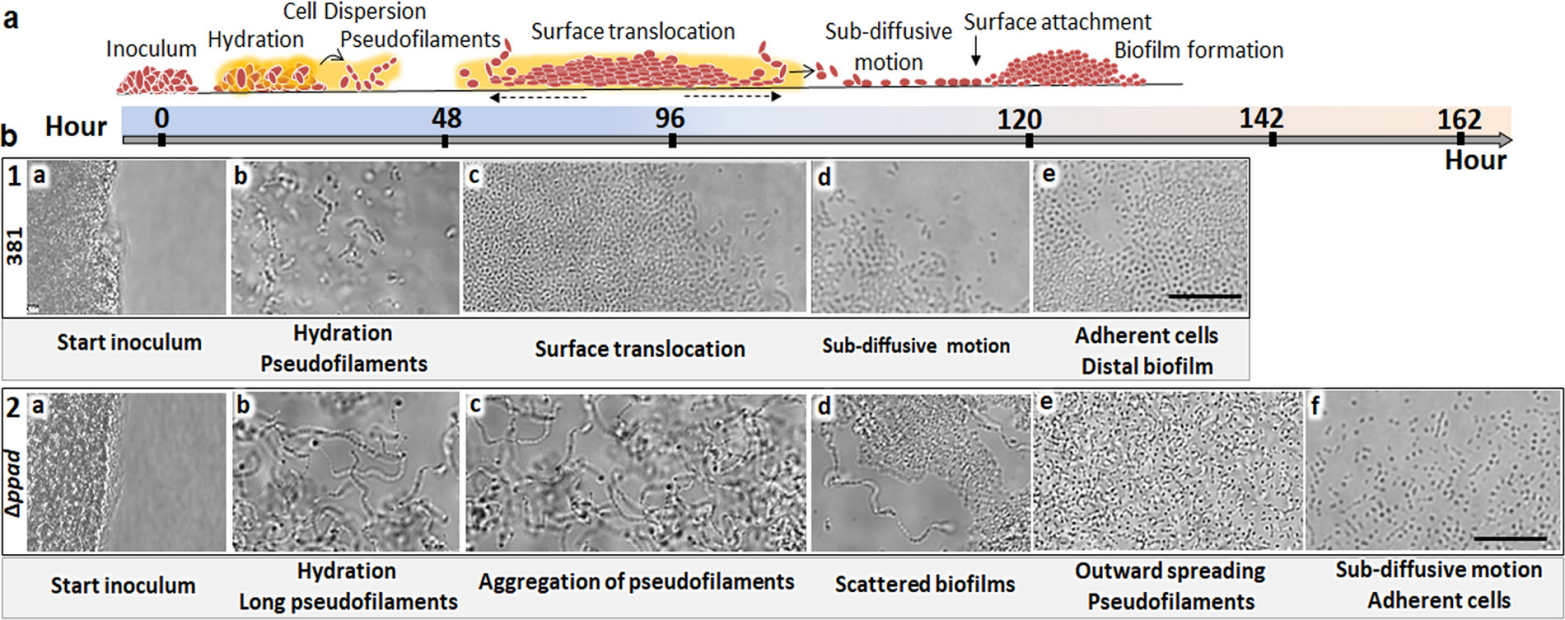
Time-lapse microscopy of surface translocation by P. gingivalis strain 381 and the Δ*ppad* mutant. (a) Schematic illustration representing sequential stages of surface translocation by reference strain 381 as recorded for more than 160 h of incubation using the chamber slide system. (b) Frames 1a to e show the chronology of surface translocation by reference strain 381, including the hydrating stage and the formation of moving pseudofilaments, outward spreading of the colony, and subdiffusive cell-driven motility by individual cells that progresses to surface translocation and the formation of distal biofilms over time. Frames 2a to f represent the recordings of disordered stages of surface translocation by strain 381Δ*ppad*. The absence of *ppad* resulted in the formation of a limited hydration zone but very long pseudofilaments whose aggregation resulted in the observation of scattered biofilms and the extension of the time required to see outward spreading of the cells and subdiffusive cell-driven motility. Scale bars: 10 μm.

Recently, the highly fimbriated P. gingivalis strain 381 (18) was shown to display a complex motility behavior when sandwiched between two surfaces, simulating the subgingival conditions (4). As shown in Fig. 1a, migration from the site of inoculation starts with the formation of a hydration zone around the inoculation site followed by the formation of pseudofilaments, outward spreading, subdiffusive cell-driven motility by individual cells, and formation of distal biofilms (4). Given that *ppad* is upregulated at initial stages of lifestyle transition and that there is enhanced biofilm growth when *ppad* is not expressed, we hypothesized that PPAD may play a key role in regulating the switch from a sessile lifestyle to surface translocation and dispersal (4, 11). In this study, the complex motility behavior of the P. gingivalis parent strain and the corresponding *ppad* mutant was comprehensively assessed using an anaerobic chamber slide system and time-lapse microscopy (4). Time-lapse recordings showed that within 24 h of incubation, the 381Δ*ppad* mutant generated a smaller hydration zone around the inoculation site than the wild-type strain (see Fig. S1 in the supplemental material). As previously defined, the hydration stage corresponds to medium digestion and the release of hygroscopic amino acids that reduce surface tension to facilitate outward migration, measured in this study as the outermost migration of cells from the initial inoculum (4). Within 48 h of time-lapse recording, the 381Δ*ppad* mutant formed very long pseudofilaments compared with the parent strain (Fig. 1b, frames 2b and 2c; see also Fig. S2). At between 48 to 96 h, when the wild-type cells displayed outward spreading and colony expansion, long pseudofilaments of the *ppad* mutants tended to aggregate in the hydrated area (see Videos S1 to S3 in the supplemental material). Compared with wild-type strain 381, which displayed subdiffusive cell-driven motility around 120 h after incubation (Video S4), aggregation of 381Δ*ppad* cells extended the period of colony expansion and delayed progression to subdiffusive cell-driven motility to about 160 h (Fig. 1b, frames 1 and 2; see also Video S5). Similarly, cryo-scanning electron microscopy (Cryo-SEM) analysis of 381 and 381Δ*ppad* colony biofilms grown on blood agar plates showed that after 4 days of growth, 381Δ*ppad* cells were unable to migrate from the colony biofilm to colonize the agar surface or form microcolonies (Fig. S3). This assessment indicates that PPAD activity plays a key role in dispersal by determining the length of pseudofilaments and facilitating colony expansion and surface translocation in strain 381.

Furthermore, when strains were stabbed to the bottom of soft agar plates and examined after 4 days, parent strain 381 migrated and attached to the polystyrene plate at the interface of the plate and soft agar, both at the site of inoculation and at distal sites, while the *ppad* deletion mutant did not migrate from the site of inoculation (Fig. S4b), suggesting that the pattern of migration by the wild-type strain is influenced by PPAD activity, consistent with our other findings (Fig. S1).

For a previous study (11), a functional copy of PPAD (PGF_00008820) was cloned into plasmid pT-COW under the control of the low-level, constitutive P. gingivalis *groES* promoter, generating plasmid pT-C8820. For overexpression analyses, pT-C8820 was transformed into parent strain 381 (strain 381 pT-C8820). Overexpression was confirmed by colorimetric assay (Fig. S5a). Strain 381 pT-C8820 formed a highly dense population of spreading pseudofilaments in the hydrated area, unlike the parent strain (381 pT-COW), which demonstrated a transition to attachment and biofilm formation during the same time frame (Fig. S5d). These data correspond to a decrease in biofilm biomass associated with 381 pT-C880 compared to 381 pT-COW (Fig. S5b). Overall, these data support the model that PPAD expression is particularly important for the initiation of surface translocation (i.e., the hydration stage), regulating the length of the pseudofilaments, and the subsequent transition from pseudofilament proliferation to surface attachment and biofilm formation in strain 381.

### Transcriptomic analysis of 381 and the 381Δ*ppad* mutant.

**FIG 2:**
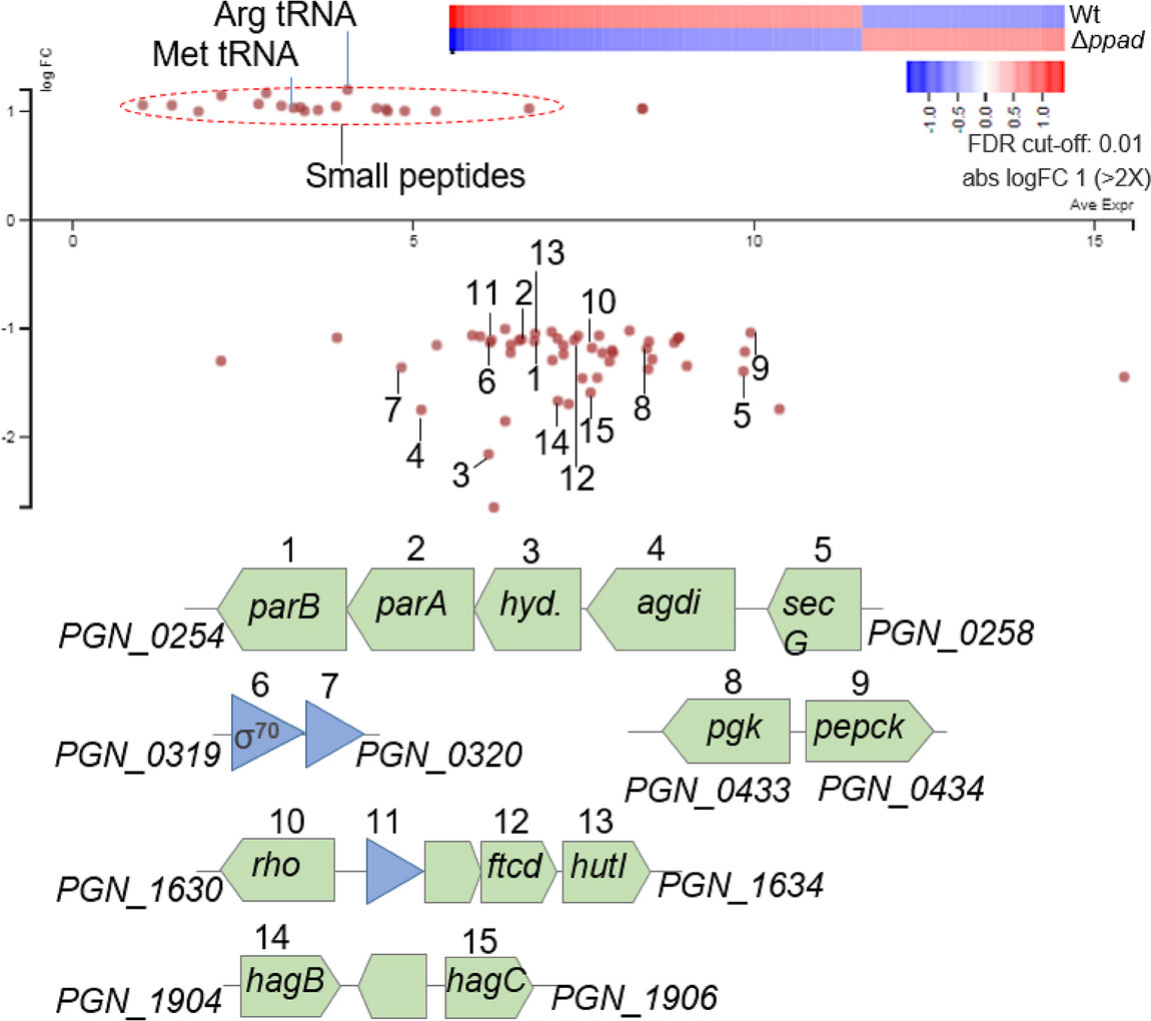
RNA-Seq analysis of strain 381 and strain 381Δ*ppad* translocating cells. (Upper panel) Differential gene expression in *ppad* deletion mutant compared with wild type (Wt) during surface translocation. FDR, false-discovery rate. (Lower panel) Downregulated genes are numbered and illustrated in operonic organizations. 3 *hyd*, gene for *N* carbamoylputrescine amidohydrolase; 4 *agdi*, gene for agmatine deiminase; 8 *pgk*, gene for phosphoglycerate kinase; 9 *pepck*, gene for phosphoenolpyruvate carboxykinase; 10 *rho*, gene for transcription termination factor Rho; 12 *ftcd*, gene for formiminotransferase-cyclodeaminase; 13 *hutI*, gene for imidazolonepropionase; 14 *hagB*, gene for HagB hemagglutinin protein; 15 *hagC*, gene for HagC hemagglutinin protein.

To determine if PPAD activity alters gene expression, we performed transcriptomic analysis on RNA extracted from cells of strains 381 and 381Δ*ppad* grown as colony biofilms on BAPHK (Northeast Laboratory Services) for 24 h (the same growth conditions as used for cryo-SEM imaging). The data in Fig. 2 (see also Table S1 in the supplemental material) represent differential gene expression (false-discovery-rate [*q*] value < 0.01) analyzed using two different programs: “Rockhopper” (19) and “Degust Web tool” (20). Transcriptomic comparison showed that only a few genomic regions were significantly downregulated in the absence of *ppad*. One such downregulated region was comprised of genes PGN_0254 to PGN_0258, which encode *N*-carbamoylputrescine amidohydrolase and agmatine deiminase (polyamine biosynthesis pathway), and *parA* and *parB* (putatively mediating cell division-dependent genome segregation) (>2-fold; *q* value < 0.01) (Fig. 2). This was concomitant with downregulation of hemagglutinin genes *hagB* and *hagC* (>2-fold; *q* value < 0.01) in the *ppad* mutant (Fig. 2). In addition, the majority of upregulated genes in the 381Δ*ppad* mutant (>2-fold; *q* value < 0.01) encoded Arg-tRNA and various hypothetical peptides that are distributed in the genome of P. gingivalis but whose functions remain unknown (Fig. 2, upper panel).

Additional experiments were run to verify transcriptome sequencing (RNA-Seq) findings of interest. Quantitative reverse transcriptase PCR (RT-qPCR) and hemagglutination assays were performed on RNA and cells from colony biofilms, respectively. N-carbamoylputrescine amidohydrolase, agmatine deiminase, hemagglutinins, and PPAD were all verified to be transcribed at lower levels in 381Δ*ppad* mutant colony biofilms than in strain 381 colony biofilms (Fig. S6a). The hemagglutination assay showed that the 381Δ*ppad* mutant exhibited a complete loss of hemagglutination at a dilution of 1:32, while strain 381 exhibited a complete loss of hemagglutination at a dilution of 1:128 (Fig. S6b). Therefore, the 381Δ*ppad* mutant showed less hemagglutination than strain 381, supporting the RNA-Seq data suggesting the presence of fewer hemagglutinins on the cell surface. These findings support the model that genes differentially expressed in the 381Δ*ppad* mutant correspond to lower levels of surface translocation and higher levels of biofilm formation.

### PPAD determines the availability of free arginine and arginine metabolism during surface translocation.

**FIG 3:**
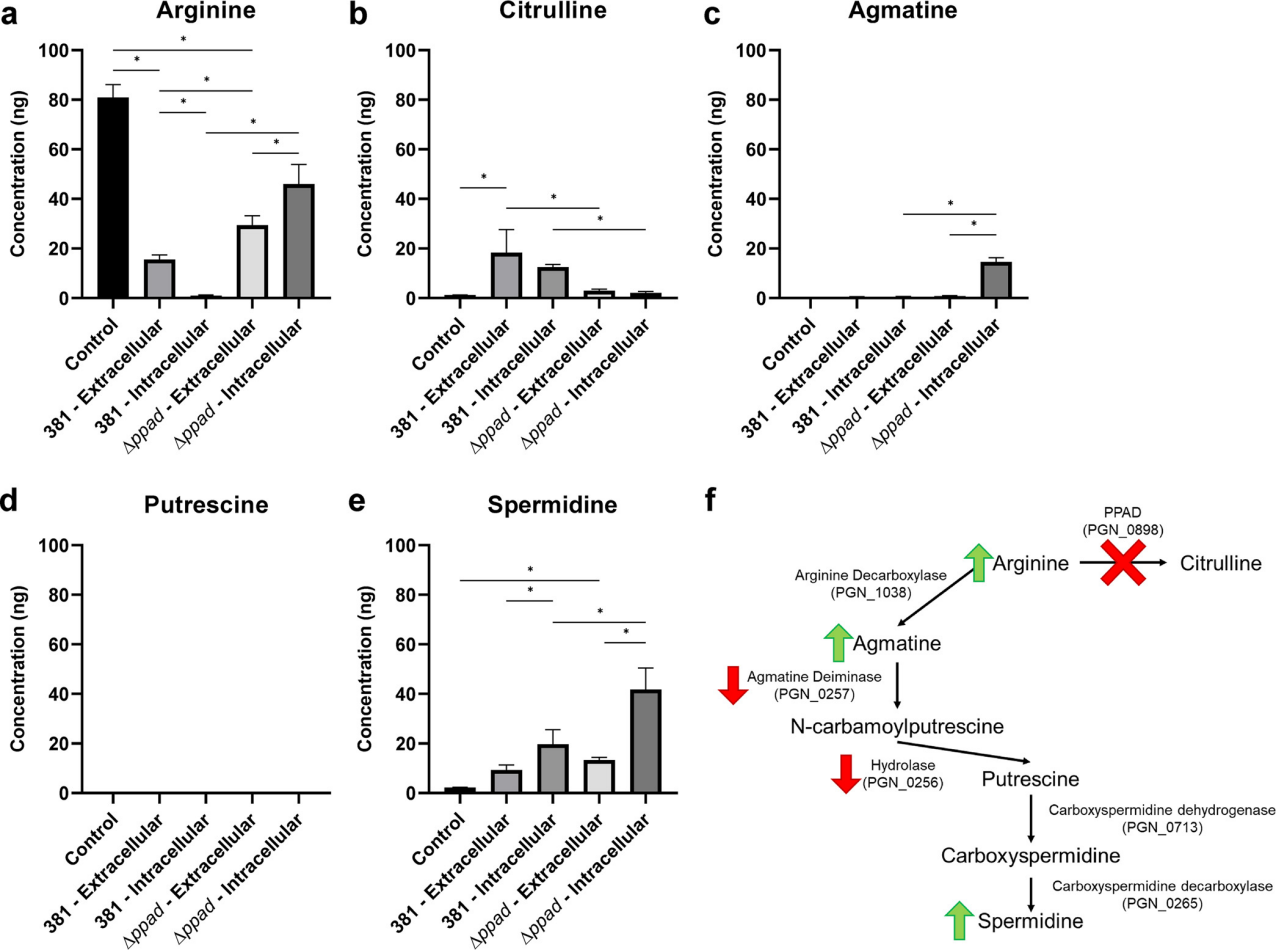
Deletion of *ppad* alters arginine metabolism. (a to e) Targeted metabolomics of intracellular and extracellular arginine and arginine-derived metabolites (a), citrulline (b), agmatine (c), putrescine (d), and spermidine (e). Strain 381 catabolizes arginine and produces measurable quantities of citrulline and spermidine. Strain 381Δ*ppad* cannot produce citrulline and instead catabolizes arginine more slowly, resulting in increased arginine availability and increased intracellular agmatine and spermidine levels. Data represent averages of results from three replicates. Error bars represent standard deviations. The data were analyzed by two-way analysis of variance (ANOVA) with Tukey’s multiple-comparison test using GraphPad Prism version 8.0.1. *, *P* < 0.05. (f) Summary of RNA-Seq and metabolomics results pertaining to arginine metabolism in the 381Δ*ppad* mutant.

As mentioned above, our transcriptomic analysis provided some insights into the correlation of PPAD function and the regulation of some genes in the pathway of arginine metabolism, specifically, the biosynthesis of polyamines. Importantly, we previously confirmed that PPAD accounts for all of the arginine deiminase activity in strain 381 (11). In order to understand the mechanism underlying the observed correlation, a metabolomic analysis targeting absolute quantification of arginine-derived metabolites was performed on the metabolomes of 381 and 381Δ*ppad* translocating cells, the extracellular milieu of surface translocating cells, and a medium-only (BAPHK) control. There was significantly more arginine in the medium control than in the extracellular milieu of 381 or 381Δ*ppad* samples, indicating that the translocating cells transported and used arginine from the environment (Fig. 3a). However, the 381Δ*ppad* samples contained ∼2 times more extracellular arginine and ∼48 times more intracellular arginine than the 381 samples (Fig. 3a). In fact, intracellular arginine was almost completely exhausted or depleted in translocating 381 samples at the time point tested (Fig. 3a). Therefore, deleting *ppad* appears to slow arginine metabolism. There was little to no citrulline in the control, indicating that any citrulline present in the experimental samples was produced by P. gingivalis (Fig. 3b). As expected, there was no citrulline produced by the 381Δ*ppad* mutant as there was no statistically significant difference between the level of citrulline in the 381Δ*ppad* samples and that seen with the control (Fig. 3b). On the other hand, strain 381, which still secreted PPAD, produced both extracellular and intracellular citrulline (Fig. 3b). With respect to the arginine-derived polyamines agmatine, putrescine, and spermidine, parent strain 381 accumulated only the end product spermidine intracellularly, which is in agreement with previous findings (Fig. 3c to e) (13). Although strain 381Δ*ppad* similarly did not accumulate putrescine, it accumulated both intracellular agmatine and spermidine (Fig. 3c to e); these data support the results of the RNA-Seq and RT-qPCR analyses, which showed less transcription of agmatine deiminase in 381Δ*ppad* samples (Fig. 2; see also Fig. S6a). Strain 381 and the 381Δ*ppad* mutant had the same levels of extracellular spermidine, but the 381Δ*ppad* mutant had ∼2 times more intracellular spermidine than strain 381 (Fig. 3e). Overall, the 381Δ*ppad* cells contained significantly more arginine, agmatine, and spermidine than the 381 cells. Our metabolomics data indicate that deleting *ppad* directly impacts arginine availability and metabolism. Rather than generating both citrulline and polyamines from arginine as in the case of strain 381, the 381Δ*ppad* mutant can generate only polyamines from arginine. As a result, arginine accumulates within 381Δ*ppad* cells, suggesting that *ppad* reduces the levels of free arginine, which is central to the physiology of P. gingivalis. Without citrullination, the presence of an excess of intracellular arginine results in increased polyamines. Counterintuitively, the intracellular concentration of the end product spermidine is higher in the 381Δ*ppad* mutant than in the 381 strain despite the downregulation of upstream genes at the time points tested (Fig. 2; see also Fig. S6a and Fig. 3e). Therefore, it is possible that an early accumulation of spermidine results in a negative-feedback loop to downregulate upstream genes in an attempt to regulate spermidine levels; however, a time course experiment measuring gene expression and metabolites would be necessary to better understand this phenomenon. Overall, our data show that the availability of free arginine is controlled by PPAD activity and that metabolic feedback imposed by the availability of arginine and/or arginine-derived metabolites may determine the direction of arginine metabolism in P. gingivalis toward citrulline production or polyamine biosynthesis.

### PPAD modulates the rate of substrate proteolysis.

**FIG 4:**
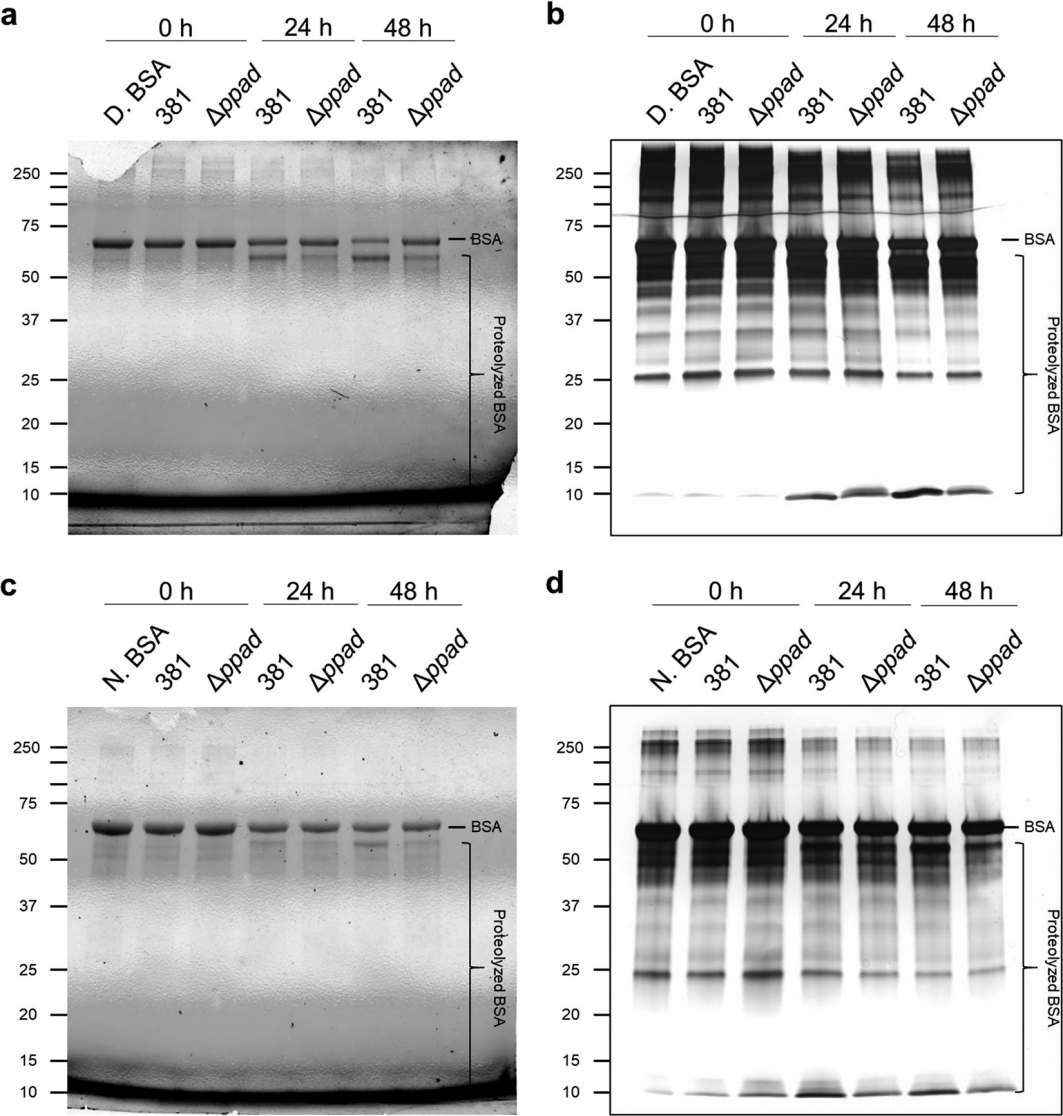
Deleting *ppad* delays the degradation of BSA. Strains 381 and 381Δ*ppad* were incubated in buffer with 1% denatured BSA (D. BSA) (a and b) or 1% native BSA (N. BSA) (c and d). Samples were taken at 0 h, 24 h, and 48 h. Samples were centrifuged to remove bacterial cells and then run on 12% Mini-Protean TGX Stain-Free protein gels (Bio-Rad). The Stain-Free gels were imaged directly (a and c) or by silver staining (b and d) using a ChemiDoc imaging system (Bio-Rad). (a) Strain 381 generated more-intense bands of degradation product than the 381Δ*ppad* mutant over time, while the band of full-length BSA became weaker. (b) Silver-stained gels showed the same overall pattern as the Stain-Free gels, but the higher sensitivity further showed greater accumulation of low-weight (∼10-kDa) degradation products in the strain 381 samples than in the mutant samples. (c and d) The 381Δ*ppad* mutant degraded native BSA more slowly than 381, but strain 381 and the 381Δ*ppad* mutant both took longer to degrade native BSA than denatured BSA.

As previously reported, the ability of P. gingivalis to generate a zone of hydration is central to the bacterium’s ability to translocate. This stage is the initial reduction of surface tension via protein hydrolysis (4). Gingipains are the predominant proteases in P. gingivalis, and they play a critical role in hydrolysis and hemolysis. Previously, Moradali et al. posited that the secretion of proteases and protein-modifying enzymes (e.g., gingipains and PPAD) is necessary for conditioning the surface as they may release hygroscopic amino acids that act as wetting agents (4). Furthermore, Vermilyea et al. showed that the 381Δ*ppad* mutant has less Rgp (Arg-gingipain) enzymatic activity in supernatant fractions due to retention or accumulation of gingipains within the biofilm matrix (11). Since we have shown that the 381Δ*ppad* mutant forms an enhanced biofilm and produces a limited hydration zone at the initial stage of surface translocation, we hypothesized that deleting *ppad* would impact the proteolysis of nutritional substrates and subsequent development of a zone of hydration. To this end, we investigated potential differences in the rate of proteolysis and consequent dispersion of proteolyzed protein between strain 381 and the 381Δ*ppad* mutant by evaluating the utilization of bovine serum albumin (BSA). Previously, we did not observe differences in growth between strain 381 and the 381Δ*ppad* mutant using rich medium (THBHK) or minimal medium (chemically defined medium supplemented with 1% tryptone [CDM-T]) that contained peptides readily utilized by P. gingivalis (11). When P. gingivalis was grown in 1% native BSA, the 381Δ*ppad* mutant exhibited a slight defect in growth, indicating that PPAD positively correlates with nutritional uptake (Fig. S7a). Further, a denatured form of BSA was incubated with strain 381 or the 381Δ*ppad* mutant for 0, 24, or 48 h in assay buffer and subjected to SDS-PAGE analysis to investigate degradation patterns using fluorescent detection of proteins in Mini-Protean TGX Stain-Free protein gels and silver-stained gels. Fluorescent detection showed that both strain 381 and the 381Δ*ppad* mutant generated a major band at the expected size of intact denatured BSA (∼66 kDa) and one major degradation product just below the full-length BSA (Fig. 4a). Over time, strain 381 generated bands of degradation product that were more intense than those generated by the 381Δ*ppad* mutant while the band of full-length BSA became weaker (Fig. 4a). Silver-stained gels showed the same overall pattern as Stain-Free gels, but the higher sensitivity further showed greater accumulation of low-weight degradation products (∼10 kDa) in the strain 381 samples than in the mutant samples (Fig. 4b). This analysis indicates that PPAD plays an important role in determining the rate of protein degradation. We further investigated the interplay of protein conformation and PPAD function by testing the rate of native BSA degradation. Once again, the 381Δ*ppad* mutant degraded native BSA more slowly than strain 381 did, but both strain 381 and the 381Δ*ppad* mutant took longer to degrade native BSA than denatured BSA (Fig. 4c and d). Analysis of both denatured and native forms of BSA showed that the 381Δ*ppad* mutant was, as expected, unable to citrullinate any of the substrates whereas the parent strain citrullinated both denatured and native BSA, which may increase the efficacy of degradation (Fig. S7b). Taken together, these findings show that the 381Δ*ppad* mutant, which is unable to citrullinate substrates and its own proteins, demonstrated reduced proteolytic activity, which in turn made the 381Δ*ppad* mutant less effective at degrading proteins for nutrients and less effective at generating a zone of hydration to initiate translocation.

### PPAD promotes the biogenesis of outer membrane vesicles and biopearling of vesicle chains and tubes.

**FIG 5:**
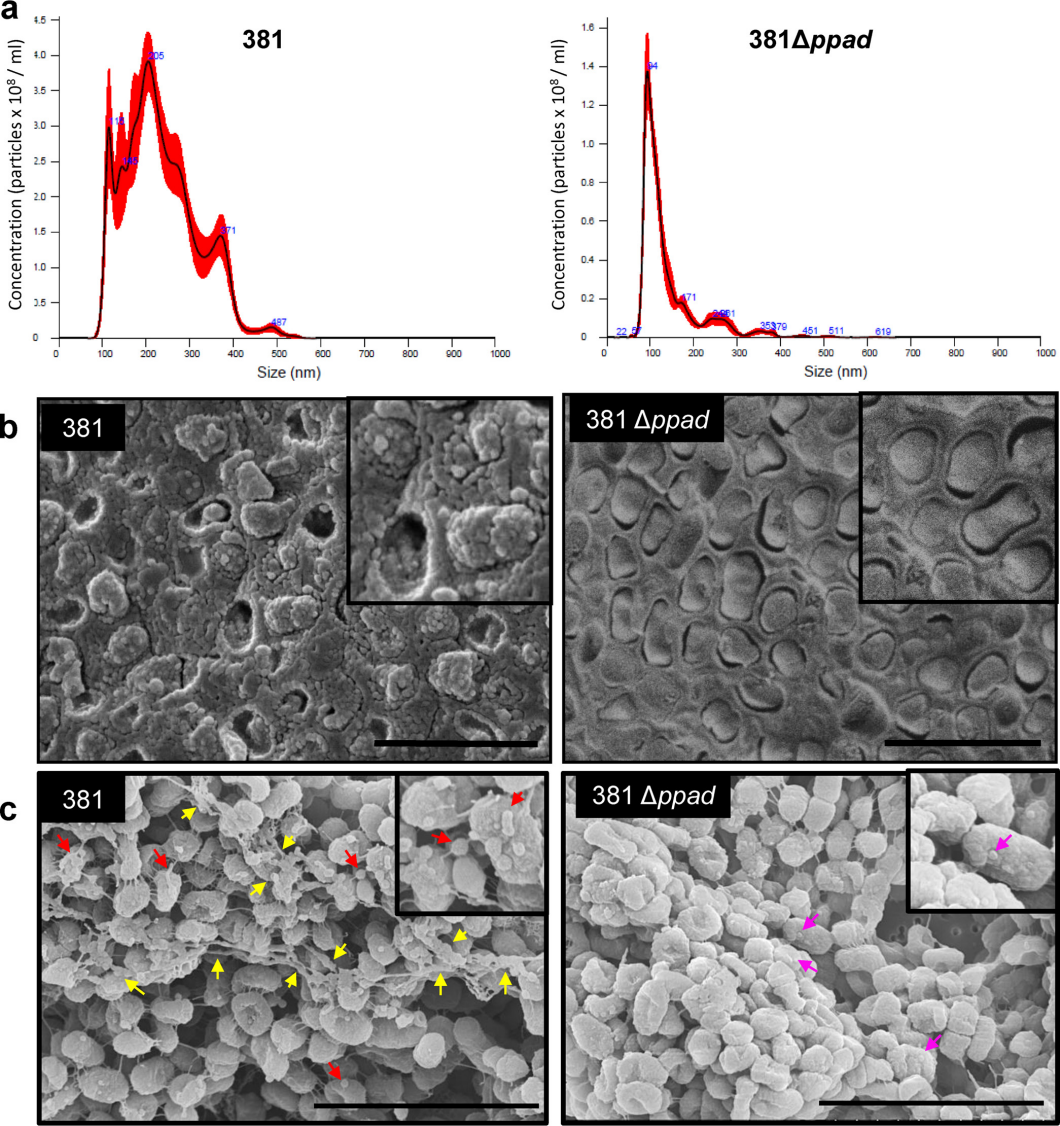
Deletion of ppad inhibits OMV biogenesis and release. (a) NanoSight analysis of crude OMV preparations from 381 and 381Δ*ppad* planktonic cultures. The 381Δ*ppad* mutant produced OMVs that were fewer and smaller in size than those produced by strain 381. (b) Cryo-SEM imaging of colony biofilms showed that the 381 *ppad* mutant produced few to no OMVs within the biofilm matrix compared to the parent strain. (Inlay) Magnified section of micrograph. Scale bar: 2 μm. (c) Formation of biopearling assemblies by strain 381. SEM imaging of fixed content of the hydration zone showed that strain 381 formed biopearling assemblies (yellow arrows) via connecting membrane protrusions and large-size OMVs (red arrows) under surface translocation conditions. Deletion of *ppad* resulted in production of few small-sized OMVs (pink arrows) and in inhibition of biogenesis of large-size OMVs and biopearling assemblies. (Inlay) Magnified section of micrograph. Scale bar: 3 μm.

In P. gingivalis, key virulence factors such as PPAD, gingipains, and adhesins are outer membrane-associated proteins secreted by the T9SS. These outer membrane-associated virulence factors are also dispersed into the surroundings upon the biogenesis and release of OMVs. Therefore, OMVs, which originate from the outer membrane through an as-yet-unknown mechanism, are heavily loaded with T9SS cargo proteins. Previous findings demonstrated that PPAD citrullinated surface- and OMV-associated proteins, including gingipain-derived adhesins, whereas PPAD absence resulted in less gingipain activity in supernatant fractions and formation of a copious amounts of cell-associated protein aggregates (11, 21). Given our findings showing that the 381Δ*ppad* mutant is deficient in the rate of proteolysis and forms a limited zone of hydration during the initial stage of surface translocation, we hypothesized that the secretion of T9SS cargo proteins via OMVs is impacted in the absence of PPAD. To test this hypothesis, we generated crude OMV preparations from planktonic cultures (500 ml) of strain 381 and the 381Δ*ppad* mutant. The crude preparations were used in order to minimize manipulation of the culture and thereby best compare and contrast the quantities and size distribution of OMVs produced by the two strains. During OMV preparation, no visible macroscopic pellet was produced by the 381Δ*ppad* mutant, while the parent strain produced a visible pellet, as usual. After suspension of the ultracentrifuged pellets, nanoparticle tracking analysis (NanoSight) was performed to evaluate size distribution. The particle size distribution pattern of the 381Δ*ppad* preparation was distinct from that of the 381 preparation. NanoSight analysis of the 381Δ*ppad* crude OMV preparation showed a predominant peak around 100 nm and an average particle size of 138.6 ± 1.2 nm (Fig. 5a). NanoSight analysis of the 381 crude OMV preparation showed a wider particle size range, with a predominant peak around 200 nm and an average particle size of 233.1 ± 2.0 nm (Fig. 5a). Furthermore, comparing the concentrations of particles of the predominant peaks alone, strain 381 produced ∼3 times more particles than the 381Δ*ppad* mutant (Fig. 5a). Overall, NanoSight analysis showed that the *ppad* mutant generated particles that were fewer and smaller than those produced by the parent strain. Further, Cryo-SEM imaging of fixed colony biofilms showed that the 381Δ*ppad* mutant produced few to no OMVs in the biofilm matrix compared to the wild-type strains (Fig. 5b). Finally, SEM imaging of the fixed content of the hydration zone during surface translocation showed that the wild-type cells produced larger numbers of membrane protrusions and OMVs which connected to each other and formed long chains of OMVs, also known as biopearling assemblies (22), which not only connected distant cells to each other but also increased surface-to-volume ratios for optimal nutrient uptake (Fig. 5c). In contrast, the 381Δ*ppad* mutant was defective in the production of membrane protrusions, large-sized OMVs, and the biopearling assemblies under surface translocation conditions (Fig. 5c). Taken together, our findings show that secretion of enzymatically active PPAD positively impacts the biogenesis of OMVs in P. gingivalis.

## DISCUSSION

**FIG 6:**
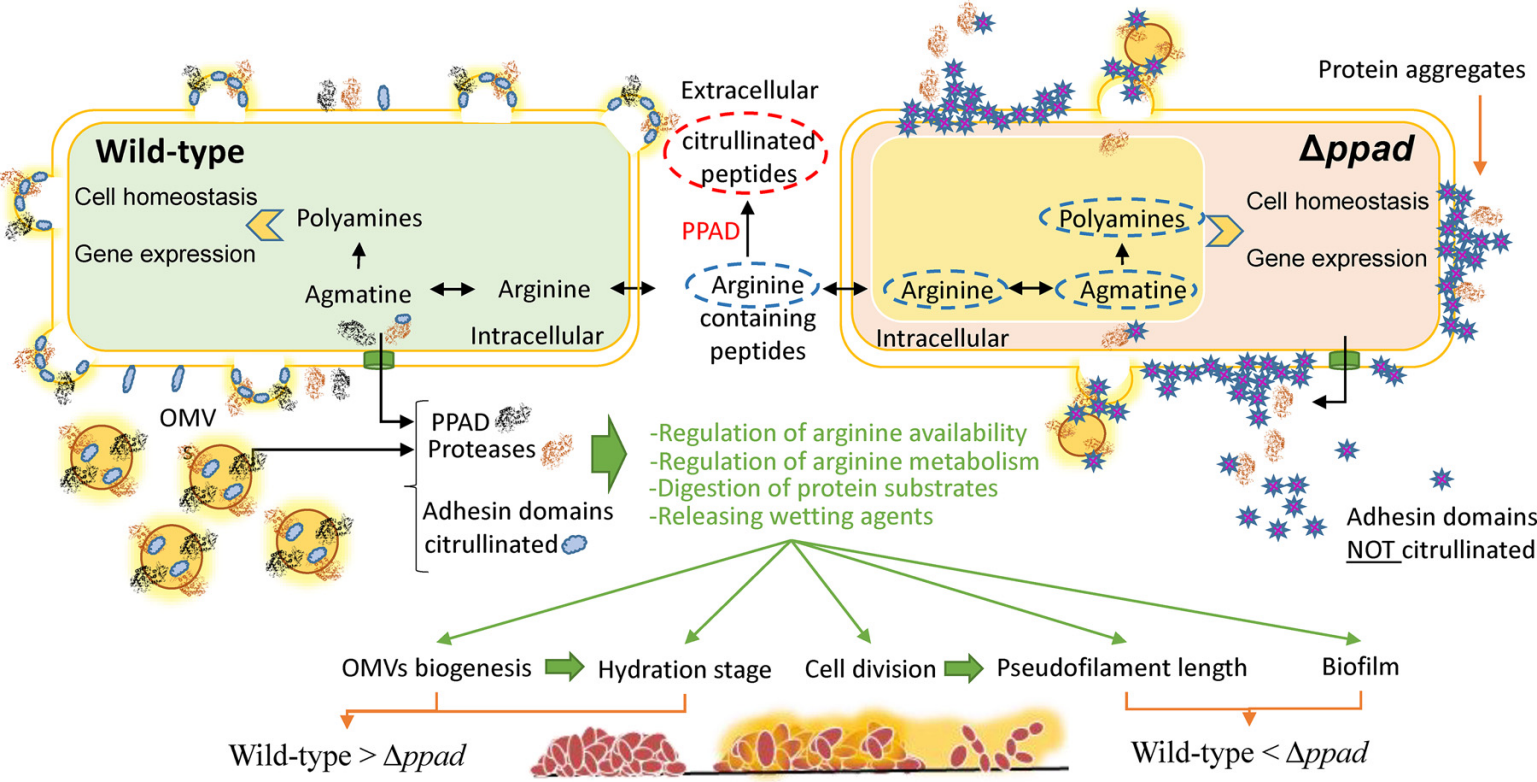
Proposed working model describing the biological role of P. gingivalis PPAD enzyme. The secretion of PPAD enzyme regulates the availability of free arginine and citrullinate surface proteins. These events are required for the optimal metabolism of arginine and arginine-derived metabolites (e.g., polyamines) and for optimal distribution and secretion of surface proteins, including proteases, particularly via facilitating biogenesis and/or secretion of OMVs. Consequently, optimal release of proteases and PPAD regulates the rate of protein digestion and nutrient release and facilitates the release of wetting agents and the formation of the hydration zone required for surface translocation. Also, metabolic-feedback loops of arginine metabolism regulate normal cell division and formation of pseudofilaments during surface translocation via regulating *par* loci.

The role of PPAD in the basic physiology of P. gingivalis has remained enigmatic until recently. Our previous work showed that when PPAD is active, this limits P. gingivalis strain 381 biofilm development, specifically, the accretion of cells and proteins within the biofilm matrix (11). We now know that P. gingivalis strain 381 is capable of a unique type of surface migration that allows cells to translocate along an interface and that *ppad* is more extensively transcribed during this process (4). Here, we further advanced our understanding of the mechanisms involved in P. gingivalis surface translocation. The data indicate that PPAD activity positively regulates OMV biogenesis and constrains biofilm development while promoting the early stages of migration. Importantly, since the substrate for PPAD is arginine, deletion of *ppad* resulted in increased arginine levels and a subsequent increase in the levels of intracellular spermidine. Overall, the data support our model that PPAD impacts the transition between sessile growth and surface translocation by modulating OMV-mediated proteolysis and arginine metabolism (Fig. 6).

Proteases such as arginine gingipains (RgpA/B) are important for generating peptides containing arginine residues, in particular, arginine residues located at the C terminus of peptides, which PPAD can then citrullinate (23, 24). Our previous study showed that there was less Rgp activity in the supernates of 381Δ*ppad* cultures, indicating less secretion of gingipains (11). In the current study, we showed that deleting *ppad* decreased overall proteolytic activity (Fig. 4) and lowered the degree of hemagglutination (see Fig. S6b in the supplemental material), two characteristics that are critical in regard to surface translocation, optimal modification of the surroundings, and development of a zone of hydration. The hydration stage is characterized by protease-mediated hydrolysis of protein substrates and accumulation of hygroscopic amino acids and citrulline (4). Unlike P. gingivalis strain W50, which was shown previously to still produce citrulline when PPAD was deleted (25), we have determined that the 381Δ*ppad* mutant lacks the ability to generate citrulline (11) and has a limited ability to generate a hydration zone within 24 h of incubation compared to its parent strain (Fig. S1). Those data further support our previous notion that cumulative actions of PPAD and proteases indicate a P. gingivalis natural moisturizing mechanism that is phylogenetically similar to those described in mammalian systems (4, 26–29). Therefore, we propose that PPAD promotes lifestyle transition by contributing to the release of wetting agents underlying a hydrating mechanism and reduction of surface tension in various biological systems.

One of the most intriguing findings from this study was production of biopearling assemblies of OMVs and membrane protrusions by strain 381 and the defect in OMV and biopearling biogenesis mediated by the PPAD mutant. OMVs play key roles in cell communication, modification of the surroundings, facilitation of the transfer of cargo substances, biofilm formation, and host-pathogen interactions (30–32). Recent publications have shown that membrane morphogenic processes mediated by marine flavobacterium species (phylogenetically related to P. gingivalis) lead to protrusion of tubes and OMVs, which then can be transformed into chains of interconnected vesicles or biopearling (22). It has been proposed that biopearling assemblies increase surface-to-volume ratios of the cells and are optimal biological platforms for presentation of a large amount of T9SS and various hydrolyzing enzymes for maximizing nutrient uptake under specific conditions (22). The notion of a link between the T9SS and OMV biogenesis is also supported by our recent discovery that a 381 ppGpp-null mutant was defective in production of OMVs and synthesis of OMV-associated T9SS proteins (33). Also, it has been shown that numerous proteins carried on P. gingivalis OMVs can be citrullinated (21). The data presented in this study show that when strain 381 cannot citrullinate peptides and/or proteins (Δ*ppad* mutant), the strain is deficient in OMV and biopearling biogenesis (Fig. 5); hence, it is tempting to speculate that citrullination of cell surface or cell wall proteins may be linked to OMV production and subsequent biopearling. This model is supported by a recent report showing that OMV/MV release by Escherichia coli and Staphylococcus aureus can be regulated by deimination/citrullination by mammalian PADs (34) and that arginine residues within the adhesin domain of the gingipains (Kgp and Rgp), which are carried on OMVs, can be citrullinated (11). That being said, studies have shown that while there are data suggesting that PPAD can citrullinate arginine residues within proteins (11, 17, 24, 35), detailed studies have determined that PPAD preferentially citrullinates C-terminal arginine residues of peptides (36); hence, whether PPAD has the ability to directly citrullinate proteins, like the mammalian PADs, is not clear. Since polyamines are key regulatory compounds, an indirect effect of PPAD activity on polyamine biosynthesis may also impact OMV biogenesis. Studies are ongoing to determine the underlying mechanism linking PPAD activity and OMV biogenesis.

RNA-Seq analysis showed that the N-carbamoylputrescine amidohydrolase gene, the agmatine deiminase gene, *parA*/*parB*, and *hagB* and/or *hagC* (duplicated gene) were all downregulated in 381Δ*ppad* cells (Fig. 2). *N*-Carbamoylputrescine amidohydrolase and agmatine deiminase are two enzymes in the CASDH/CASDC polyamine biosynthesis pathway which results in the production of spermidine in P. gingivalis (Fig. 3f) (12, 13). However, the role of polyamines such as spermidine in P. gingivalis physiology is not clear. Spermidine regulates several processes in bacteria such as growth, biofilm formation, and swarming motility (15). Polyamines are able to modulate various processes, in part, because they are positively charged, which allows them to bind to nucleic acids, influencing characteristics such as DNA condensation and gene expression (37). Here, we show that deletion of *ppad* impacts development of a hydration zone and that arginine, agmatine, and spermidine levels are higher in 381Δ*ppad* cells, which are unable to produce citrulline or translocate effectively. Therefore, accumulation of polyamines such as spermidine can be a way for P. gingivalis to sense and respond to extracellular and/or intracellular arginine levels and to regulate the transition between surface translocation and biofilm formation.

As noted above, genes predicted to encode ParA (PGN_0255) and ParB (PGN_0254) were downregulated in the *ppad* deletion mutant. In many bacterial species, homologs to these genes (48% and 41% identity to Mycobacterium smegmatis *parA* and *parB*, respectively) coordinate chromosome segregation with cell elongation and division (38). The downregulation of *parA* and *parB* is consistent with our time-lapse imaging of surface translocating cells, which demonstrated that the *ppad* deletion mutant formed longer pseudofilaments than the parent strain. Therefore, we propose that the downregulation of the putative *parA* and *parB* genes may cause insufficient or impaired genome segregation between dividing cells, favoring the formation of longer cellular chains, and that cellular levels of arginine and/or arginine-derived metabolites may play a regulatory role in genome segregation. In addition, it was previously shown that cooperative and collective cell-on-cell rolling induced a specific movement to the pseudofilament structures, resulting in forward displacements within the hydrated area, resembling a wriggling motion (4). Therefore, another mechanism that may impact the length of the filaments is a defect in cell-on-cell rolling. Although we observed that this type of movement was reduced, it was not consistent or clear if formation of longer filaments caused less movement or if the reduction in the level of movement was the result of the longer cellular structures. Hence, it is not yet clear if PPAD plays a role in this cell-on-cell rolling.

As for *hagB* and *hagC*, these two genes encode identical hemagglutinins which are outer membrane proteins that bind red blood cells, leading to hemagglutination, which in turn facilitates iron acquisition and heme accumulation on the cell surface (39). Therefore, downregulation of hemagglutinins helps to explain the delayed pigmentation phenotype in the *ppad* deletion mutant as observed by our group and others (11, 40). HagB and HagC are also important for biofilm formation and attachment to host cells (41–43). Given the enhanced biofilm phenotype of the 381Δ*ppad* mutant, lower expression of *hagB* and *hagC* is counterintuitive; however, the 381Δ*ppad* mutant still produces the long fimbriae, the short fimbriae, and gingipain-derived hemagglutinins and adhesins, as well as other Hag proteins, all of which play a role in biofilm development. Additionally, Hag-deficient mutants have been shown to compensate by upregulating gingipains RgpA and Kgp (41). RgpA and Kgp were not upregulated in the 381Δ*ppad* mutant, but Rgp and Kgp activity is higher in 381Δ*ppad* biofilms (11). Therefore, downregulation of Hag proteins may be linked to the accumulation of gingipains in 381Δ*ppad* biofilms. Furthermore, the downregulation of *hagB* and/or *hagC* may help to explain differences in P. gingivalis attachment and invasion of host cells. Specifically, a *ppad* deletion mutant in fimbriated strain 33277 was shown to attach to and invade gingival fibroblasts less than the parent strain (44).

As periodontal disease progresses, P. gingivalis and OMV-associated virulence factors (e.g., PPAD and gingipains) may disseminate through the bloodstream, which may explain frequently reported correlations between P. gingivalis and systemic diseases. The study of P. gingivalis and PPAD function has specifically been linked to the development and/or progression of rheumatoid arthritis, while the study of P. gingivalis and gingipains has gained notoriety recently due to a reported link to Alzheimer’s disease (45–47). Therefore, our findings showing that PPAD activity impacts either directly or indirectly the release of gingipain-containing OMVs and gingipain-mediated proteolysis may make PPAD an appealing therapeutic target for not only rheumatoid arthritis but other systemic diseases such as Alzheimer’s disease. In conclusion, the results of our study demonstrate that PPAD impacts key features of P. gingivalis physiology, including proteolysis, OMV biogenesis, biofilm formation, and surface translocation.

## MATERIALS AND METHODS

### Bacterial strains and culture conditions.

The bacterial strains used in this study are listed in Table S2 in the supplemental material. P. gingivalis strains and derivatives were grown on Trypticase soy agar plates supplemented with 5 μg ml^−1^ hemin, 1 μg ml^−1^ menadione, and 5% defibrinated sheep blood (BAPHK) (Northeast Laboratory Services) at 37°C in an anaerobic chamber (Coy Lab Products) with an atmosphere containing 5% hydrogen, 10% carbon dioxide, and 85% nitrogen. Planktonic cultures of P. gingivalis were grown in Todd-Hewitt broth (Becton, Dickinson and Company), or in Trypticase soy broth (Becton, Dickinson and Company) where indicated, supplemented with 5 μg ml^−1^ hemin and 1 μg ml^−1^ menadione (THBHK or TSBHK, respectively). P. gingivalis deletion mutants were verified out of the freezer stock by supplementing media with 10 μg ml^−1^ erythromycin and were then regrown on plates or in broth without antibiotics for all subsequent assays. For P. gingivalis strains grown as colony biofilms, P. gingivalis was grown anaerobically in THBHK for 24 h at 37°C, subcultured into prereduced THBHK, and grown overnight. Cultures were diluted to an optical density at 600 nm (OD_600_) of 1.0 and diluted 1:10, and then aliquots (10 μl) were spotted onto BAPHK. Plates were grown anaerobically for 4 to 6 days.

### Construction and complementation of PPAD mutant strain.

Deletion and replacement of the entire coding region of the gene encoding PPAD (PGF_00008820) with *ermF* were performed for a previous study (11). Complementation of the PPAD deletion mutant (Δ8820) in *trans* was verified by inserting a functional copy of PGF_00008820 into plasmid pT-COW under the control of the low-level, constitutive P. gingivalis *groES* promoter, generating plasmid pT-C8820 (11). Plasmid pT-C8820 was transformed into the Δ8820 mutant by electroporation. For control strains, plasmid pT-COW was transformed into both parent strain 381 and the Δ8820 mutant by electroporation.

### PPAD enzymatic activity.

Stationary-phase cultures were diluted to the same OD_600_, and the PPAD enzymatic activity assay was set up in a 96-well PCR plate (Bio-Rad Laboratories, Inc.) and performed as previously described (48). In brief, 10 μl of culture was added to 35 μl of incubation buffer (0.1 M Tris-HCl buffer [pH 7.5], 5 mM dithiothreitol [DTT]) for experimental wells or to 40 μl of incubation buffer for control wells. A 5-μl volume of substrate (BAEE [*N*α-benzoyl-l-arginine ethyl ester hydrochloride], denatured BSA, or native BSA) was added to each experimental well to reach a final concentration of 5 mM. A 5-μl volume of substrate was added to 45 μl of incubation buffer for substrate-only controls. The plate was incubated at 37°C in a thermocycler for 30 min. A 150-μl volume of freshly prepared citrulline detection reagent {1 volume of solution A [80 mM 2,3-butanedione monoxime and 2 mM thiosemicarbazide] and 3 volumes of solution B [3 M H_3_PO_4_, 6 M H_2_SO_4_, and 2 mM NH_4_Fe(SO_4_)_2_·12·H_2_O]} was added to each well, and then the plate was incubated at 95°C in a thermocycler for 15 min. The samples were then transferred to a 96-well flat-bottom plate (Corning, Inc.), and enzymatically produced citrulline was detected at an absorbance of 540 nm.

### Biofilm assay.

Biofilm assays in uncoated 96-well polystyrene plates were performed in a chemically defined medium supplemented with 1% tryptone (CDM-T) as previously described (49). In brief, P. gingivalis was grown for 24 h in THBHK. Cultures were suspended and then diluted to an OD_600_ of 0.2 in CDM-T. A 200-μl volume was added to each well of a 96-well flat-bottom plate. Plates were then incubated for 24 h under anaerobic conditions. Plates were then washed twice by immersion in deionized water and dried. Biomass was then stained with 100 μl 0.1% safranin for 15 min. Plates were washed twice by immersion in deionized water and dried. Safranin was solubilized in 200 μl 90% ethanol (EtOH)–1% SDS for 15 min. A 150-μl volume of solubilized safranin was transferred to a new 96-well plate. Absorbance was measured at 492 nm.

### Time-lapse microscopy imaging.

Time-lapse microscopy of translocating cells was performed as previously described (4). In brief, chamber slides were filled with soft agar medium (Todd-Hewitt broth–0.3% agar) and the medium was allowed to solidify. A coverslip inoculated with a tiny dot of cells at the center was inverted and placed onto the chamber filled with medium and mounted with nail polish. Imaging was performed at the interface of agar medium and coverslip. Phase-contrast microscopy and time-lapse imaging were performed using an inverted Nikon Eclipse Ti microscope system (Nikon, Tokyo, Japan) equipped with a motorized stage (Nikon), an Andor Zyla 5.5 scientific complementary metal oxide semiconductor (sCMOS) camera, a Perfect Focus system, and automated controls (NIS-Elements; Nikon). The microscope was located inside a Coy anaerobic chamber under the conditions described above. Using a Nikon 100× 1.40-numerical-aperture (NA) lens objective, surface translocation was monitored and recorded every minute for 7 to 10 days and every 15 ms for 2 to 3 min for recording fast movements.

### Macroscopic visualization of surface translocation and zone of hydration.

To macroscopically visualize surface translocation at the interface of the polystyrene plate and soft agar layer, P. gingivalis was stabbed through a soft agar layer of BAPHK in order to deposit cells at the bottom of the plate on the polystyrene plate surface, as previously described (4). At least three replicates were used for each set of samples.

### Cryo-SEM of colony biofilms.

Electron microscopy and image analysis were performed by staff members of the Electron Microscopy Core of the Interdisciplinary Center for Biotechnology Research (ICBR) at the University of Florida (UF). Cryo-SEM experiments were performed using a Quorum PP3010T cryotransfer system (Quorum Technologies, Electron Microscopy Sciences) attached to a Hitachi SU5000FE variable-pressure scanning electron microscope (VP-SEM) (Hitachi High Technologies, USA). Samples were transported to the UF ICBR EM Core under anaerobic conditions using AnaeroPack sachets (Thermo Scientific) inside Kapak SealPAK pouches (VWR) and were then heat sealed. Colonies were immediately fixed by immersion with 4% formaldehyde–1% glutaraldehyde–0.1 M cacodylate buffer (pH 7.24) and kept at 4°C overnight. Samples were prepared for Cryo-SEM by removal of the colony biofilm from the blood agar substrate with a no. 11 scalpel and mounted onto a specimen shuttle containing a carbon adhesive tab (Electron Microscopy Sciences) on an aluminum stub. The sample containing the shuttle was attached to a PrepDek workstation transfer rod device, plunged frozen into liquid nitrogen slush at −210°C under vacuum conditions, and quickly transferred to the Cryo-prep chamber. To remove any condensed ice from the surface gained during transfer, the sample temperature was raised to −95°C and the sample was sublimed for 10 min. To avoid charging artifacts and to render the sample conductive, a thin layer of platinum was sputter coated for 60 s at 10 mA current in an argon atmosphere at −95°C. The Cryo-prep chamber was returned to −195°C and to a vacuum greater than 1,000 to 500 Pa and transferred to the nitrogen gas-cooled cold stage inside the SEM chamber. The sample remained frozen during the imaging at −195°C under high-vacuum conditions that included 5 to 6 keV, current emission of 176,000 nA, and a working distance of between 5 and 10 mm.

### SEM of surface translocating cells.

Imaging of surface translocating cells was performed by SEM. The P. gingivalis parent strain or the *Δppad* mutant growing under surface translocation conditions was prepared as described previously (4). After removal of the soft agar layer, 20 ml of phosphate-buffered saline (PBS) was added to the surrounding area of an outwardly spreading colony to collect surface translocating cells and transferred onto the surface of an Isopore 100-nm-pore-size polycarbonate filter (Millipore, Burlington, MA) pretreated with poly-l-lysine (Sigma) (0.01% in water). The filter paper was left to be partly air dried and was then laid on top of a filter pad (by stacking multiple layers of normal filter papers) soaked with fixative solution in a small petri dish to allow diffusion of fixative buffer to the sample. Subsequent 0.1 M cacodylate washes, a graded ethanol series dehydration, and drying with hexamethyldisilazane (Electron Microscopy Sciences, Hatfield, PA) were carried out by changing the filter pad beneath the sample-containing polycarbonate filter. The dried sample was mounted onto a carbon adhesive tab on an aluminum stub and sputter coated with Au/Pd in an argon-filled chamber (DeskV; Denton Vacuum, Moorestown, NJ) and examined, and digital micrographs were acquired with a field-emission scanning electron microscope (SU-5000; Hitachi High Technologies, America, Inc., Schaumburg, IL).

### Proteolytic activity.

P. gingivalis was grown in TSBHK overnight. Samples were diluted to an OD_600_ of 0.3 in basal salt (BS) buffer (14 mM Na_2_HPO_4_, 10 mM KCl, 10 mM MgCl_2_, pH 7.3) plus cysteine. Samples were centrifuged at 4,255 × *g* for 20 min at 4°C. Supernatants were decanted, and the pellets were resuspended in BS buffer to a final OD_600_ of 0.3. A 950-μl volume of P. gingivalis was added to 50 μl of 20% BSA–BS buffer–cysteine. An aliquot was immediately removed and centrifuged to remove bacterial cells (0 h). The remaining sample was incubated at 37°C with shaking. Aliquots were removed at 24 h and 48 h and centrifuged to remove bacterial cells. Samples were diluted 1:32 in Laemmli buffer (Bio-Rad) and heated at 95°C for 5 min. Ten microliters was loaded loaded per well of 12% Mini-Protean TGX Stain-Free protein gels (Bio-Rad). The gels were run at 150 V until the samples reached the bottom of the gels. The Stain-Free gels were imaged directly or after silver staining using a ChemiDoc imaging system (Bio-Rad).

### Quantification of OMVs.

For OMV isolation, P. gingivalis was grown in TSBHK to late exponential phase (OD_600_ of 1.0). Cells were removed by centrifugation at 8,000 × *g* for 30 min at 4°C. The collected supernatant (500 ml) was filtered through a 0.22-μm-pore-size filter and then concentrated through a 100-kDa filter using a Minimate tangential-flow filtration (TFF) system (Pall Life Sciences, Victoria, Australia) according to the manufacturer’s instructions. The collected concentrate was centrifuged at 100,000 × *g* for 2 h at 4°C to yield a crude OMV preparation. The pellet was then resuspended in 800 μl HEPES buffer (50 mM HEPES, 150 mM NaCl, pH 6.8). The size distribution and concentration of particles in the OMV preparations were determined using a Malvern NanoSight NS300 instrument.

### RNA-Seq.

RNA extraction for RNA-seq and RT-qPCR analyses were performed in the anaerobic chamber to avoid aerobic stress using a Direct-zol RNA miniprep kit (Zymo Research) as previously described (4, 50, 51). Downstream processing for preparing RNA samples was conducted at the Gene Expression & Genotyping Core of the Interdisciplinary Center for Biotechnology Research (ICBR), University of Florida. Quality control (QC) of RNA samples was performed using a Qubit 2.0 fluorometer, and RNA quality was assessed by applying an Agilent 2100 bioanalyzer. RNA-Seq library construction was performed for samples with calculated values of 28S/18S ratios greater than 1.0 and RNA integrity number (RIN) equal or greater than 7.0. Ribosomal RNAs (rRNAs) were eliminated from 600 ng of total RNA using an Illumina Ribo-Zero magnetic kit for bacterial RNA according to the manufacturer’s protocol. Yielded depleted RNAs were used for library construction by utilizing a NEBNext Ultra RNA library prep kit for Illumina and according to the manufacturer's user guide. Briefly, 5 μl of purified product was fragmented using the first-strand synthesis reaction buffer mix by heating at 94°C for 8 min followed by first-strand cDNA synthesis using reverse transcriptase and random primers. Synthesis of double-stranded cDNA was conducted using the second-strand master mix provided in the kit. The resulting cDNA was end repaired, subjected to dA tailing, and ligated with NEBNext adaptors. Finally, the library was enriched by PCR amplification and purified by the use of an Agencourt AMPure XP system (Beckman Coulter). For the quality control of the library and pooling, barcoded libraries were sized on the bioanalyzer and then quantitated by the use of Qubit assay kits (Invitrogen). Typically, a 200-to-1,000 broad library peak value was observed. Quantitative PCR was used to validate the library's functionality, using Kapa library quantification kits for Illumina platforms (Kapa Biosystems, catalog no. KK4824). All samples were subjected to equimolar pooling for one lane of a HiSeq 3000 run of 2 × 100 cycles. Sequencing was performed on the Illumina HiSeq 3000 system instrument using the clustering and sequencing reagents provided by Illumina. Paired-end runs of 2 × 100 cycles required the adding together of reagents from the 150-cycle and the 50-cycle kits (catalog no. FC-410-1002, FC-410-1001, and PE-410-1001). Sequencing reactions were set up using 5 μl of the library (2.5 nM). The program “Rockhopper” (19) was used for aligning sequencing reads to the genome references (i.e., P. gingivalis 381), assembling transcripts, identifying their boundaries and constructing transcriptome maps, quantifying transcript abundance, data normalizing, and testing for differential gene expression (*q* value < 0.01). The Degust Web tool (20) was also applied for visualizing differential gene expression. Identification of differentially expressed genes and determination of operonic organization and possible cognate metabolic and nonmetabolic cellular processes were conducted using various bioinformatics databases, including mainly KEGG (52), BioCyc (53, 54), and the National Center for Biotechnology Information (NCBI) databases (https://www.ncbi.nlm.nih.gov).

### RT-qPCR.

RNA was extracted from 1-day-old colony biofilms using a Direct-zol RNA miniprep kit (Zymo Research). cDNA was generated from 2.5 ng RNA using RNA to cDNA EcoDry premix (Clontech). For relative quantification of desired genes, RT-qPCR was conducted in a total volume of 20 μl containing 1 μl of 1:10 diluted cDNA, a 0.5 μM concentration of each primer (Table S3), 6 μl of PCR-grade water, and 10 μl of 2× iQ SYBR green supermix. Amplification and detection of product were performed using a CFX96 Touch real-time PCR detection system (Bio-Rad), and the cycling conditions were as follows: 95°C for 3 min and then 39 cycles of 95°C for 20 s, 55°C for 20 s, and 72°C for 20 s. Fluorescence was detected after each cycle. In each experiment, the target and control samples were amplified in the same plate, and the experiments were conducted in triplicate and normalized internally using the average cycle quantification (*C_q_*) value for the reference gene (16S). To confirm the specificity of the amplified products, automated melting curve analysis was performed. All primers used in this study are listed in Table S3.

### Hemagglutination assay.

P. gingivalis was grown to an OD_600_ of about 1.0 in THBHK and then diluted to 1.0 and to 1:10, and 10-μl aliquots were spotted onto BAPHK. Plates were grown for 2 days. Colony biofilms were scraped from the plate and suspended in PBS, centrifuged, and then washed two more times with PBS. Samples were then resuspended in PBS at an OD_600_ of 2.0. Defibrinated sheep erythrocytes were centrifuged at 400 × *g* for 5 min at 4°C, washed twice with PBS, and resuspended to 2% defibrinated sheep erythrocytes–PBS. The bacterial suspensions were then diluted in a 2-fold series with PBS, 100 μl from each bacterial suspension was added to a round-bottom 96-well plate, and an equal volume of 2% sheep erythrocyte suspension was added to the bacterial suspensions and mixed. The plate was incubated at room temperature for 3 h.

### Targeted metabolomics.

P. gingivalis was stabbed to the bottom of BAPHK containing 0.3% agar and grown anaerobically for 3 days. Blood agar was removed, and then PBS (1 ml) was added to the petri dish. Cells were collected with scraping, and samples (4 biological replicates) were pooled and centrifuged. Washed plates with no inoculum (medium control) were collected in the same manner. Pooled supernatants (4 ml) were transferred to new tubes. Cell pellets were frozen and stored at −80°C. Supernatants (extracellular milieu) were lyophilized and stored at −80°C. Overall, for each strain there were 3 replicates for cells and 3 replicates for supernatants and each replicate included the pooled cells or supernatants from 4 plates. Samples were delivered to the Southeast Center for Integrated Metabolomics at the University of Florida for metabolomics analysis. The two internal standards (IS) used in the extraction were arginine ^15^N_2_ and ornithine d7. The IS stock mix was prepared with the resulting concentrations of arginine ^15^N_2_ (4.16 μg/ml) and ornithine d7 (4.32 μg/ml). The working solution was made by diluting the stock solution 1:4 for resulting concentrations of 1.04 μg/ml and 1.08 μg/ml, respectively.

Cell pellets were gently suspended in 40 mM ammonium formate–water. The cells were then homogenized on a BeadBeater twice for 30 s each time at 1,800 rpm in 50 μl of 5 mM ammonium acetate–water plus 10 to 20 0.7-mm-diameter zirconia beads with 10-min rests on ice between homogenization steps. The lyophilized supernatants (approximately 10 mg) were placed into 1.5-ml Eppendorf tubes. A 50-μl volume of 5 mM ammonium acetate–water was added to each sample based on the previous test and sample set and homogenized once for 30 s at 1,800 rpm on the BeadBeater with 10-min rests on ice afterward.

Protein quantitation was performed on the QuBit fluorometer, and samples were normalized to 300 μg/ml for a 25-μl extraction. All samples were extracted with 20 μl of the working IS solution and 250 μl of 80% methanol–water and incubated for 20 min at 4°C. The samples were centrifuged at 20,000 × *g* for 10 min at 4°C. A 250-μl volume of supernatant was dried under nitrogen gas at 30°C and then stored at −80°C.

The samples were reconstituted in 25 μl of 0.1% formic acid–water. Samples were then analyzed on the Thermo Q Exactive high-resolution mass spectrometer coupled to a Dionex Ultimate 3000 ultra-high-pressure liquid chromatography system. A Red Cross plasma-positive control and an extraction blank negative control were extracted as QCs. A neat QC was made with 1:1:3 metabolomics IS mix–amino acid mix–0.1% formic acid–water. Four unlabeled standards (agmatine, arginine, citrulline, and ornithine) were made at 10 μg/ml separately and vialed for analysis alongside the samples and QCs.

### Data availability.

We declare that the data supporting the findings of this study are available within the paper and its supplemental material files. Raw sequencing data are available on the NCBI Sequence Read Archive (SRA) under accession number PRJNA607521.

## Supplementary Material

Supplemental file 1

Supplemental file 2

Supplemental file 3

Supplemental file 4

supplemental file 5

Supplemental file 6

## References

[1] Moradali MF, Ghods S, Rehm BH 2017 *Pseudomonas aeruginosa* lifestyle: a paradigm for adaptation, survival, and persistence. Front Cell Infect Microbiol 7:39. doi:10.3389/fcimb.2017.00039. 28261568 PMC5310132

[2] Rossi E, Paroni M, Landini P 2018 Biofilm and motility in response to environmental and host-related signals in Gram negative opportunistic pathogens. J Appl Microbiol 125:1587–1602. doi:10.1111/jam.14089. 30153375

[3] Verstraeten N, Braeken K, Debkumari B, Fauvart M, Fransaer J, Vermant J, Michiels J 2008 Living on a surface: swarming and biofilm formation. Trends Microbiol 16:496–506. doi:10.1016/j.tim.2008.07.004. 18775660

[4] Moradali MF, Ghods S, Angelini TE, Davey ME 2019 Amino acids as wetting agents: surface translocation by *Porphyromonas gingivalis*. ISME J 13:1560–1574. doi:10.1038/s41396-019-0360-9. 30783212 PMC6775972

[5] Johnston JJ, Shrivastava A, McBride MJ 2017 Untangling Flavobacterium johnsoniae gliding motility and protein secretion. J Bacteriol 200:e00362-17. doi:10.1128/JB.00362-17. 29109184 PMC5738736

[6] Kita D, Shibata S, Kikuchi Y, Kokubu E, Nakayama K, Saito A, Ishihara K 2016 Involvement of the type IX secretion system in Capnocytophaga ochracea gliding motility and biofilm formation. Appl Environ Microbiol 82:1756–1766. doi:10.1128/AEM.03452-15. 26729712 PMC4784043

[7] Kulkarni SS, Johnston JJ, Zhu Y, Hying ZT, McBride MJ 2019 The carboxy-terminal region of Flavobacterium johnsoniae SprB facilitates its secretion by the type IX secretion system and propulsion by the gliding motility machinery. J Bacteriol 201:e00218-19. doi:10.1128/JB.00218-19. 31262839 PMC6755757

[8] McBride MJ, Nakane D 2015 Flavobacterium gliding motility and the type IX secretion system. Curr Opin Microbiol 28:72–77. doi:10.1016/j.mib.2015.07.016. 26461123

[9] Lasica AM, Ksiazek M, Madej M, Potempa J 2017 The type IX secretion system (T9SS): highlights and recent insights into its structure and function. Front Cell Infect Microbiol 7:215. doi:10.3389/fcimb.2017.00215. 28603700 PMC5445135

[10] Dashper SG, Cross KJ, Slakeski N, Lissel P, Aulakh P, Moore C, Reynolds EC 2004 Hemoglobin hydrolysis and heme acquisition by *Porphyromonas gingivalis*. Oral Microbiol Immunol 19:50–56. doi:10.1046/j.0902-0055.2003.00113.x. 14678474

[11] Vermilyea DM, Ottenberg GK, Davey ME 2019 Citrullination mediated by PPAD constrains biofilm formation in P gingivalis strain 381. NPJ Biofilms Microbiomes 5:7. doi:10.1038/s41522-019-0081-x. 32029738 PMC6367333

[12] Hanfrey CC, Pearson BM, Hazeldine S, Lee J, Gaskin DJ, Woster PM, Phillips MA, Michael AJ 2011 Alternative spermidine biosynthetic route is critical for growth of Campylobacter jejuni and is the dominant polyamine pathway in human gut microbiota. J Biol Chem 286:43301–43312. doi:10.1074/jbc.M111.307835. 22025614 PMC3234850

[13] Hosoya R, Hamana K 2004 Distribution of two triamines, spermidine and homospermidine, and an aromatic amine, 2-phenylethylamine, within the phylum Bacteroidetes. J Gen Appl Microbiol 50:255–260. doi:10.2323/jgam.50.255. 15747230

[14] Burrell M, Hanfrey CC, Murray EJ, Stanley-Wall NR, Michael AJ 2010 Evolution and multiplicity of arginine decarboxylases in polyamine biosynthesis and essential role in Bacillus subtilis biofilm formation. J Biol Chem 285:39224–39238. doi:10.1074/jbc.M110.163154. 20876533 PMC2998088

[15] Michael AJ 2018 Polyamine function in archaea and bacteria. J Biol Chem 293:18693–18701. doi:10.1074/jbc.TM118.005670. 30254075 PMC6290158

[16] Kurihara S, Suzuki H, Tsuboi Y, Benno Y 2009 Dependence of swarming in Escherichia coli K-12 on spermidine and the spermidine importer. FEMS Microbiol Lett 294:97–101. doi:10.1111/j.1574-6968.2009.01552.x. 19493013

[17] Abdullah SN, Farmer EA, Spargo L, Logan R, Gully N 2013 *Porphyromonas gingivalis* peptidylarginine deiminase substrate specificity. Anaerobe 23:102–108. doi:10.1016/j.anaerobe.2013.07.001. 23856045

[18] Arai M, Hamada N, Umemoto T 2000 Purification and characterization of a novel secondary fimbrial protein from *Porphyromonas gingivalis* strain 381. FEMS Microbiol Lett 193:75–81. doi:10.1111/j.1574-6968.2000.tb09405.x. 11094282

[19] McClure R, Balasubramanian D, Sun Y, Bobrovskyy M, Sumby P, Genco CA, Vanderpool CK, Tjaden B 2013 Computational analysis of bacterial RNA-Seq data. Nucleic Acids Res 41:e140. doi:10.1093/nar/gkt444. 23716638 PMC3737546

[20] Powell D 2019 Degust: interactive RNA-seq analysis. https://zenodo.org/record/3501067#.X70bXGVKiUs.

[21] Larsen DN, Mikkelsen CE, Kierkegaard M, Bereta GP, Nowakowska Z, Kaczmarek JZ, Potempa J, Hojrup P 2020 Citrullinome of *Porphyromonas gingivalis* outer membrane vesicles: confident identification of citrullinated peptides. Mol Cell Proteomics 19:167–180. doi:10.1074/mcp.RA119.001700. PMC6944236 31754044

[22] Fischer T, Schorb M, Reintjes G, Kolovou A, Santarella-Mellwig R, Markert S, Rhiel E, Littmann S, Becher D, Schweder T, Harder J 2019 Biopearling of interconnected outer membrane vesicle chains by a marine flavobacterium. Appl Environ Microbiol 85:e00829-19. doi:10.1128/AEM.00829-19. 31324630 PMC6752029

[23] McGraw WT, Potempa J, Farley D, Travis J 1999 Purification, characterization, and sequence analysis of a potential virulence factor from *Porphyromonas gingivalis*, peptidylarginine deiminase. Infect Immun 67:3248–3256. doi:10.1128/IAI.67.7.3248-3256.1999. 10377098 PMC116503

[24] Rodriguez SB, Stitt BL, Ash DE 2009 Expression of peptidylarginine deiminase from *Porphyromonas gingivalis* in *Escherichia coli*: enzyme purification and characterization. Arch Biochem Biophys 488:14–22. doi:10.1016/j.abb.2009.06.010. 19545534 PMC2752837

[25] Munoz-Atienza E, Flak MB, Sirr J, Paramonov NA, Aduse-Opoku J, Pitzalis C, Curtis MA 2020 The P. gingivalis autocitrullinome is not a target for ACPA in early rheumatoid arthritis. J Dent Res 99:456–462. doi:10.1177/0022034519898144. 31905316 PMC7088229

[26] Senshu T, Akiyama K, Kan S, Asaga H, Ishigami A, Manabe M 1995 Detection of deiminated proteins in rat skin: probing with a monospecific antibody after modification of citrulline residues. J Invest Dermatol 105:163–169. doi:10.1111/1523-1747.ep12317070. 7543546

[27] Stoltze L, Schirle M, Schwarz G, Schröter C, Thompson MW, Hersh LB, Kalbacher H, Stevanovic S, Rammensee HG, Schild H 2000 Two new proteases in the MHC class I processing pathway. Nat Immunol 1:413–418. doi:10.1038/80852. 11062501

[28] Takahashi M, Tezuka T 2004 The content of free amino acids in the stratum corneum is increased in senile xerosis. Arch Dermatol Res 295:448–452. doi:10.1007/s00403-003-0448-x. 14762669

[29] Kamata Y, Taniguchi A, Yamamoto M, Nomura J, Ishihara K, Takahara H, Hibino T, Takeda A 2009 Neutral cysteine protease bleomycin hydrolase is essential for the breakdown of deiminated filaggrin into amino acids. J Biol Chem 284:12829–12836. doi:10.1074/jbc.M807908200. 19286660 PMC2676013

[30] Dorward DW, Garon CF 1990 DNA is packaged within membrane-derived vesicles of Gram-negative but not Gram-positive bacteria. Appl Environ Microbiol 56:1960–1962. doi:10.1128/AEM.56.6.1960-1962.1990. 16348232 PMC184538

[31] Ellis TN, Kuehn MJ 2010 Virulence and immunomodulatory roles of bacterial outer membrane vesicles. Microbiol Mol Biol Rev 74:81–94. doi:10.1128/MMBR.00031-09. 20197500 PMC2832350

[32] Gabarrini G, Palma Medina LM, Stobernack T, Prins RC, Du Teil Espina M, Kuipers J, Chlebowicz MA, Rossen JWA, van Winkelhoff AJ, van Dijl JM 2018 There's no place like OM: vesicular sorting and secretion of the peptidylarginine deiminase of *Porphyromonas gingivalis*. Virulence 9:456–464. doi:10.1080/21505594.2017.1421827. 29505395 PMC5955434

[33] Kim HM, Davey ME 2020 Synthesis of ppGpp impacts type IX secretion and biofilm matrix formation in *Porphyromonas gingivalis*. NPJ Biofilms Microbiomes 6:5. doi:10.1038/s41522-020-0115-4. 32005827 PMC6994654

[34] Kosgodage US, Matewele P, Mastroianni G, Kraev I, Brotherton D, Awamaria B, Nicholas AP, Lange S, Inal JM 2019 Peptidylarginine deiminase inhibitors reduce bacterial membrane vesicle release and sensitize bacteria to antibiotic treatment. Front Cell Infect Microbiol 9:227. doi:10.3389/fcimb.2019.00227. 31316918 PMC6610471

[35] Stobernack T, Glasner C, Junker S, Gabarrini G, de Smit M, de Jong A, Otto A, Becher D, van Winkelhoff AJ, van Dijl JM 2016 The extracellular proteome and citrullinome of the oral pathogen *Porphyromonas gingivalis*. J Proteome Res 15:4532–4543. doi:10.1021/acs.jproteome.6b00634. 27712078

[36] Goulas T, Mizgalska D, Garcia-Ferrer I, Kantyka T, Guevara T, Szmigielski B, Sroka A, Millán C, Usón I, Veillard F, Potempa B, Mydel P, Solà M, Potempa J, Gomis-Rüth FX 2015 Structure and mechanism of a bacterial host-protein citrullinating virulence factor, *Porphyromonas gingivalis* peptidylarginine deiminase. Sci Rep 5:11969. doi:10.1038/srep11969. 26132828 PMC4487231

[37] Childs AC, Mehta DJ, Gerner EW 2003 Polyamine-dependent gene expression. Cell Mol Life Sci 60:1394–1406. doi:10.1007/s00018-003-2332-4. 12943227 PMC11138590

[38] Ginda K, Santi I, Bousbaine D, Zakrzewska-Czerwińska J, Jakimowicz D, McKinney J 2017 The studies of ParA and ParB dynamics reveal asymmetry of chromosome segregation in mycobacteria. Mol Microbiol 105:453–468. doi:10.1111/mmi.13712. 28517109

[39] Lepine G, Ellen RP, Progulske-Fox A 1996 Construction and preliminary characterization of three hemagglutinin mutants of *Porphyromonas gingivalis*. Infect Immun 64:1467–1472. doi:10.1128/IAI.64.4.1467-1472.1996. 8606121 PMC173946

[40] Gully N, Bright R, Marino V, Marchant C, Cantley M, Haynes D, Butler C, Dashper S, Reynolds E, Bartold M 2014 *Porphyromonas gingivalis* peptidylarginine deiminase, a key contributor in the pathogenesis of experimental periodontal disease and experimental arthritis. PLoS One 9:e100838. doi:10.1371/journal.pone.0100838. 24959715 PMC4069180

[41] Connolly E, Millhouse E, Doyle R, Culshaw S, Ramage G, Moran GP 2017 The *Porphyromonas gingivalis* hemagglutinins HagB and HagC are major mediators of adhesion and biofilm formation. Mol Oral Microbiol 32:35–47. doi:10.1111/omi.12151. 28051836

[42] Song H, Bélanger M, Whitlock J, Kozarov E, Progulske-Fox A 2005 Hemagglutinin B is involved in the adherence of *Porphyromonas gingivalis* to human coronary artery endothelial cells. Infect Immun 73:7267–7273. doi:10.1128/IAI.73.11.7267-7273.2005. 16239522 PMC1273858

[43] Mendez KN, Hoare A, Soto C, Bugueño I, Olivera M, Meneses C, Pérez-Donoso JM, Castro-Nallar E, Bravo D 2019 Variability in genomic and virulent properties of *Porphyromonas gingivalis* strains isolated from healthy and severe chronic periodontitis individuals. Front Cell Infect Microbiol 9:246. doi:10.3389/fcimb.2019.00246. 31355151 PMC6635597

[44] Gawron K, Bereta G, Nowakowska Z, Lazarz-Bartyzel K, Lazarz M, Szmigielski B, Mizgalska D, Buda A, Koziel J, Oruba Z, Chomyszyn-Gajewska M, Potempa J 2014 Peptidylarginine deiminase from *Porphyromonas gingivalis* contributes to infection of gingival fibroblasts and induction of prostaglandin E2 -signaling pathway. Mol Oral Microbiol 29:321–332. doi:10.1111/omi.12081. 25176110 PMC4617314

[45] Potempa J, Mydel P, Koziel J 2017 The case for periodontitis in the pathogenesis of rheumatoid arthritis. Nat Rev Rheumatol 13:606–620. doi:10.1038/nrrheum.2017.132. 28835673

[46] Olsen I, Singhrao SK, Potempa J 2018 Citrullination as a plausible link to periodontitis, rheumatoid arthritis, atherosclerosis and Alzheimer's disease. J Oral Microbiol 10:1487742. doi:10.1080/20002297.2018.1487742. 29963294 PMC6022223

[47] Dominy SS, Lynch C, Ermini F, Benedyk M, Marczyk A, Konradi A, Nguyen M, Haditsch U, Raha D, Griffin C, Holsinger LJ, Arastu-Kapur S, Kaba S, Lee A, Ryder MI, Potempa B, Mydel P, Hellvard A, Adamowicz K, Hasturk H, Walker GD, Reynolds EC, Faull RLM, Curtis MA, Dragunow M, Potempa J 2019 *Porphyromonas gingivalis* in Alzheimer's disease brains: evidence for disease causation and treatment with small-molecule inhibitors. Sci Adv 5:eaau3333. doi:10.1126/sciadv.aau3333. 30746447 PMC6357742

[48] Knipp M, Vasák M 2000 A colorimetric 96-well microtiter plate assay for the determination of enzymatically formed citrulline. Anal Biochem 286:257–264. doi:10.1006/abio.2000.4805. 11067748

[49] Christopher AB, Arndt A, Cugini C, Davey ME 2010 A streptococcal effector protein that inhibits *Porphyromonas gingivalis* biofilm development. Microbiology (Reading) 156:3469–3477. doi:10.1099/mic.0.042671-0. 20705665 PMC7336479

[50] Moye ZD, Gormley CM, Davey ME 2018 Galactose Impacts the Size and Intracellular Composition of the Asaccharolytic Oral Pathobiont *Porphyromonas gingivalis*. Appl Environ Microbiol 85:e02268-18. doi:10.1128/AEM.02268-18. PMC6365826 30552185

[51] Rocha FG, Moye ZD, Ottenberg G, Tang P, Campopiano DJ, Gibson FC III, Davey ME 2020 *Porphyromonas gingivalis* sphingolipid synthesis limits the host inflammatory response. J Dent Res 99:568–576. doi:10.1177/0022034520908784. 32105543 PMC7174802

[52] Kanehisa M, Furumichi M, Tanabe M, Sato Y, Morishima K 2017 KEGG: new perspectives on genomes, pathways, diseases and drugs. Nucleic Acids Res 45:D353–D361. doi:10.1093/nar/gkw1092. 27899662 PMC5210567

[53] Caspi R, Billington R, Ferrer L, Foerster H, Fulcher CA, Keseler IM, Kothari A, Krummenacker M, Latendresse M, Mueller LA, Ong Q, Paley S, Subhraveti P, Weaver DS, Karp PD 2016 The MetaCyc database of metabolic pathways and enzymes and the BioCyc collection of pathway/genome databases. Nucleic Acids Res 44:D471–D480. doi:10.1093/nar/gkv1164. 26527732 PMC4702838

[54] Caspi R, Altman T, Billington R, Dreher K, Foerster H, Fulcher CA, Holland TA, Keseler IM, Kothari A, Kubo A, Krummenacker M, Latendresse M, Mueller LA, Ong Q, Paley S, Subhraveti P, Weaver DS, Weerasinghe D, Zhang P, Karp PD 2014 The MetaCyc database of metabolic pathways and enzymes and the BioCyc collection of Pathway/Genome Databases. Nucleic Acids Res 42:D459–D471. doi:10.1093/nar/gkt1103. 24225315 PMC3964957

