# Supplementary material for: PPAD Activity Promotes Outer Membrane Vesicle Biogenesis and Surface Translocation by Porphyromonas gingivalis
Source: J Bacteriol. 2021 Jan 25;203(4):e00343-20. doi: 10.1128/JB.00343-20 (PMC7847538; doi:10.1128/JB.00343-20)
Supplement: Supplemental file 6 [file Figures.pdf]

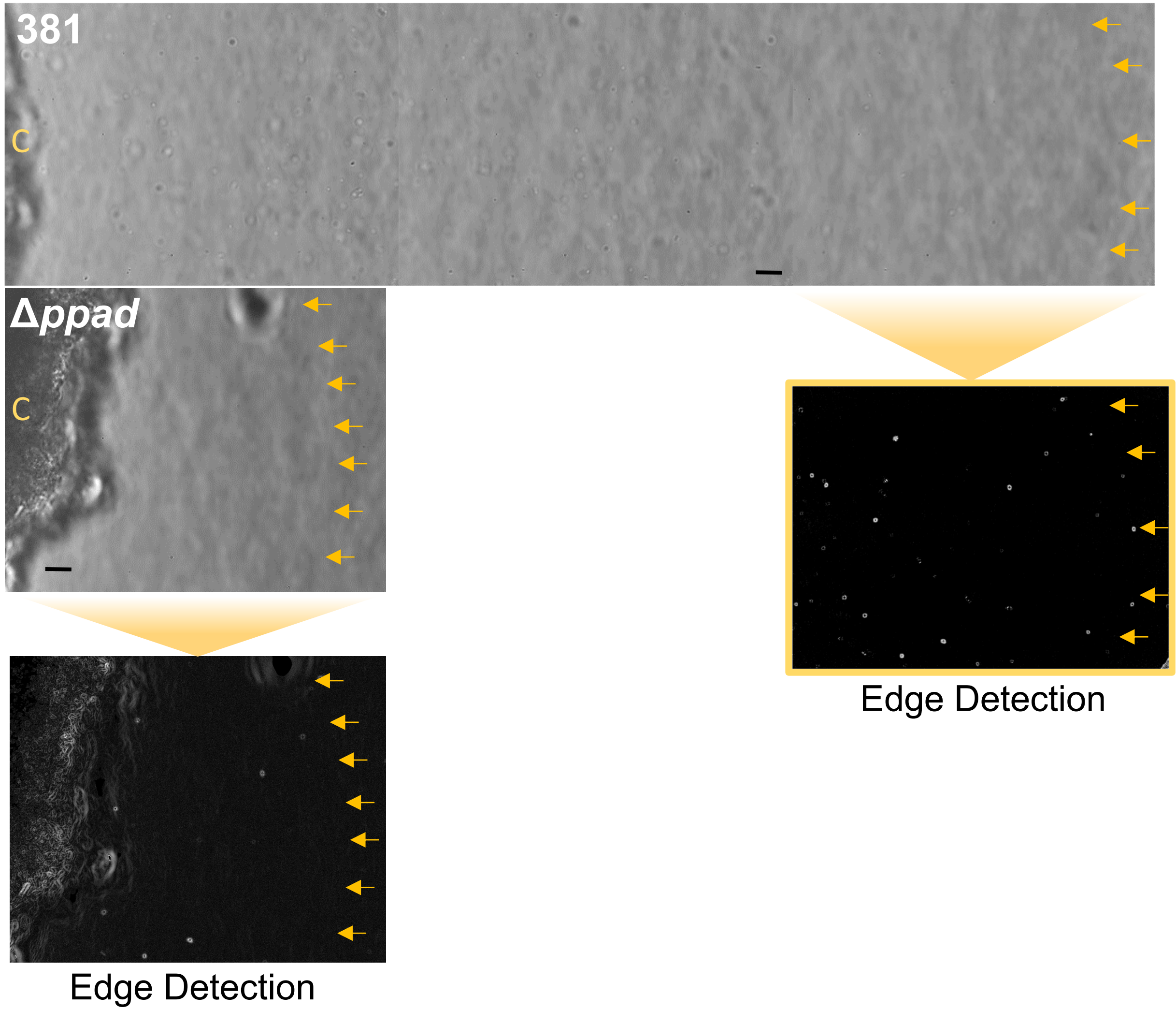


**Supplemental Fig. 1** Hydration zone formed by strain 381 and 381Δ*ppad* mutant within 24 h of incubation in the chamber slide that contains suspending cells – yellow arrows represents frontline of the hydration zone and edge detection frames show floating cells (white dots) in the hydrated area and indicate the edge of the hydration zoon as opposed to non-hydrated area without suspending cells. Due to difficulty in distinguishing the edge of the hydrated areas in 2-dimentional format, NIS-Elements-Detect Edges was used to filter not only background noise but also to retain the images' details i.e. suspending cells as an indictor of the hydrated area. C, stabbed site. Scale bar: 10 µm

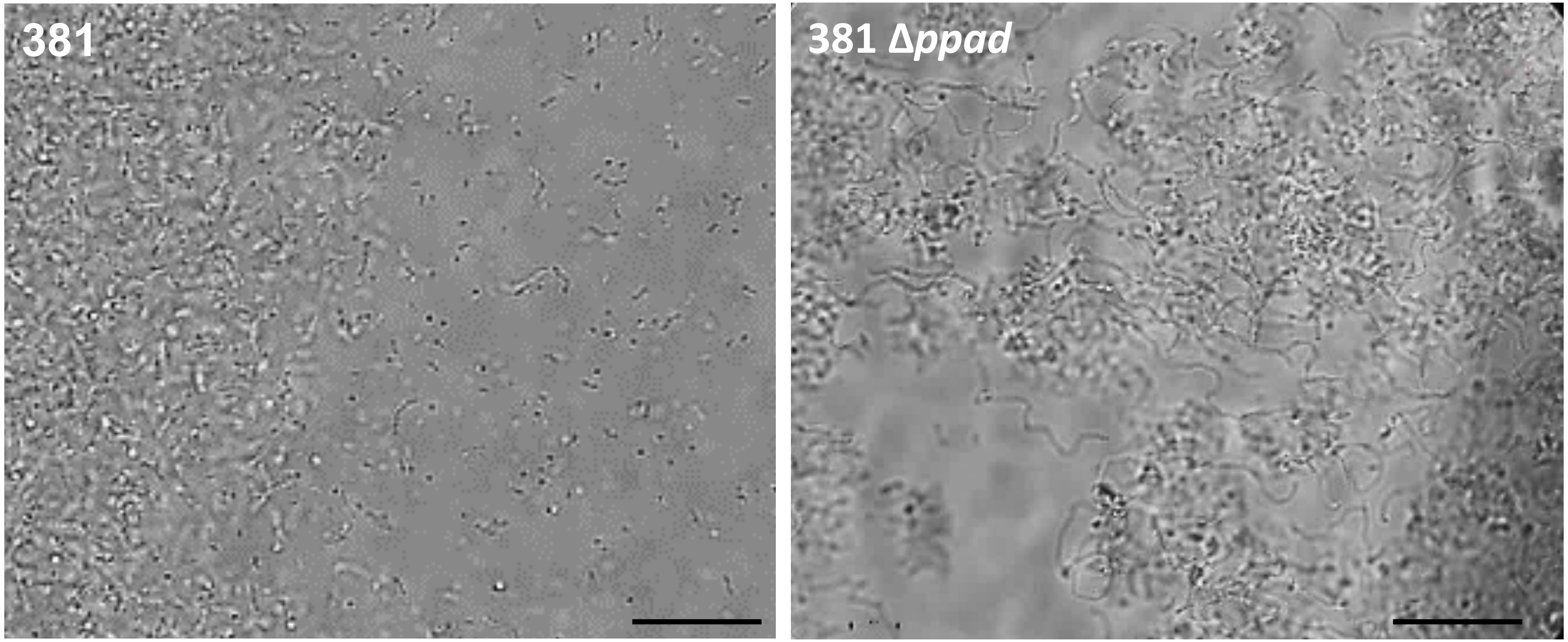


**Supplemental Fig. 2** Deletion of *ppad* in strains 381 resulted in the formation of long pseudofilaments.
Scale: 10 μm

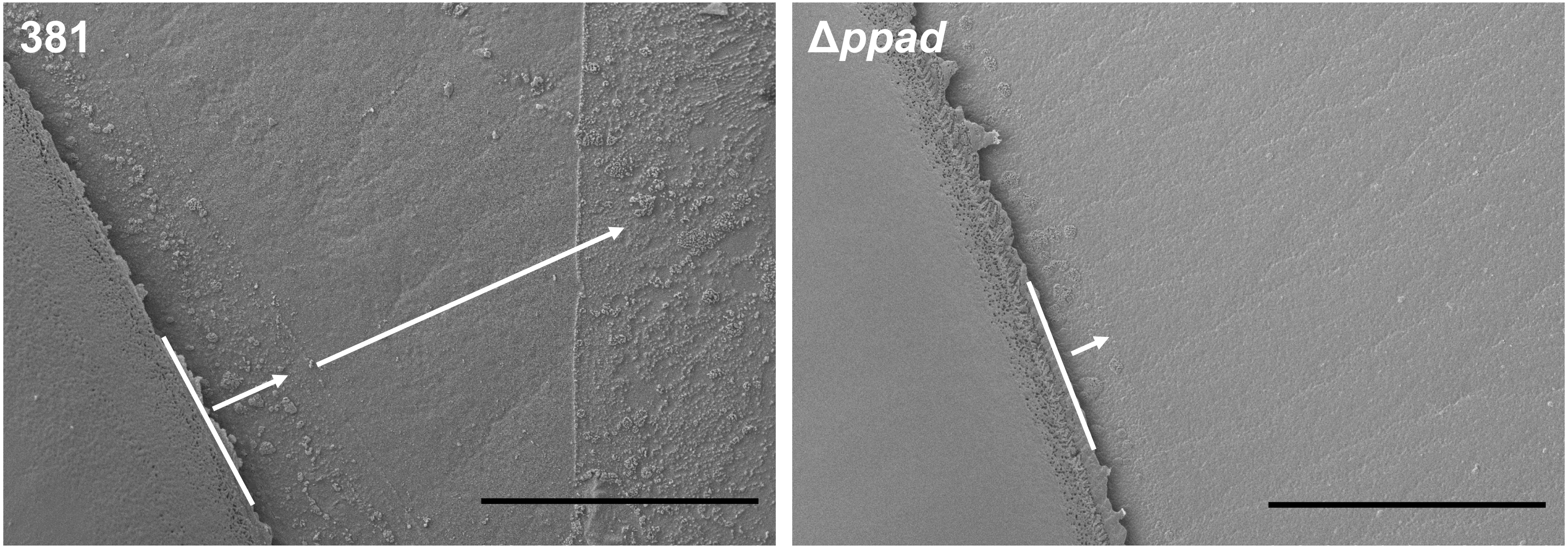


**Supplemental Fig. 3** Deletion of *ppad* inhibits dispersal from colony biofilms. Colony biofilms were grown on BAPHK and imaged by Cryo-SEM. White lines indicate the edge of the colony biofilm. White arrows indicate dispersal from the colony biofilm. Parent strain 381 dispersed from the colony biofilm and formed microcolonies at distant sites. Individual cells were observed between the colony biofilm and the microcolonies upon magnification. Δ*ppad* was not able to disperse from the colony biofilm and individual cells were not observed on the agar surface around the colony biofilm. Scale: 300 µm

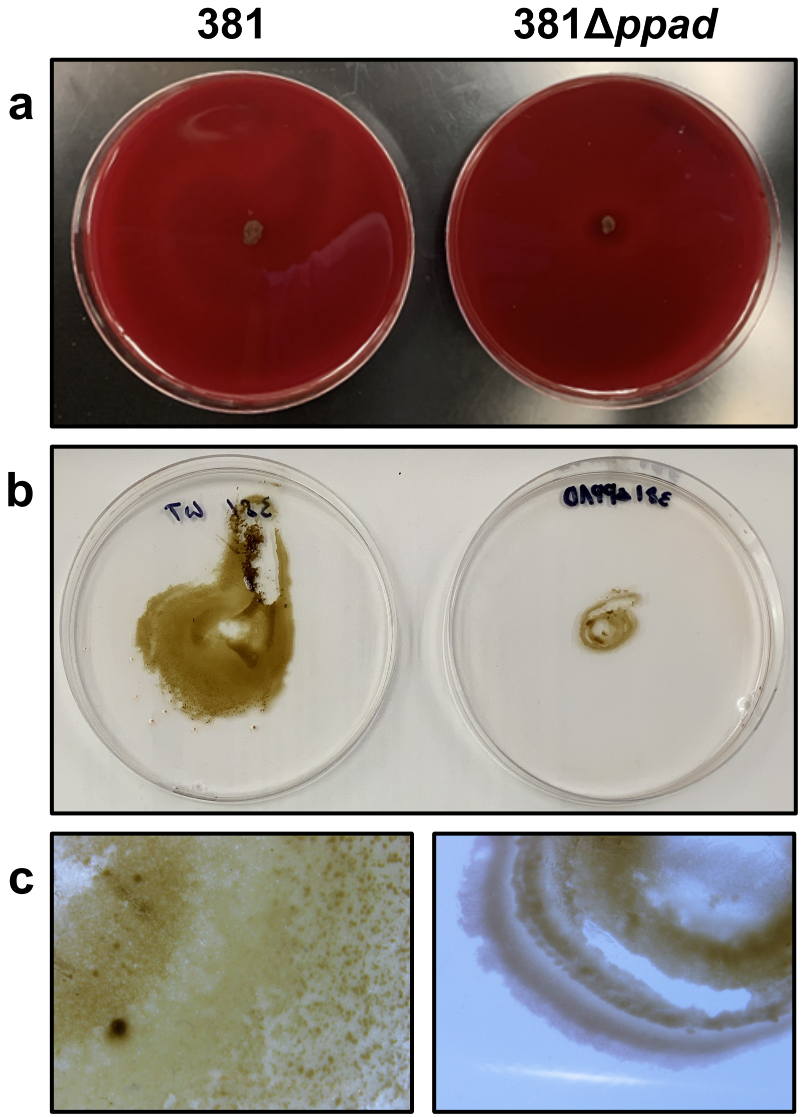


**Supplemental Fig. 4** Deleting *ppad* inhibits migration. 381 and the 381Δ*ppad* mutant were stabbed to the bottom of soft agar plates and grown for 4 days anaerobically. (**a**, ) Top-down view of inoculation site after 4 days. (**b**) *P. gingivalis* cells attached to the polystyrene plate after removal of blood agar and washing. 381 attached to the polystyrene plate both at the site of inoculation and at distal sites. 381Δ*ppad* did not migrate from the site of inoculation. (**c**) Magnified images from the edges of surface-attached cells showing the formation of satellite microcolonies by 381, but not by 381Δ*ppad*.

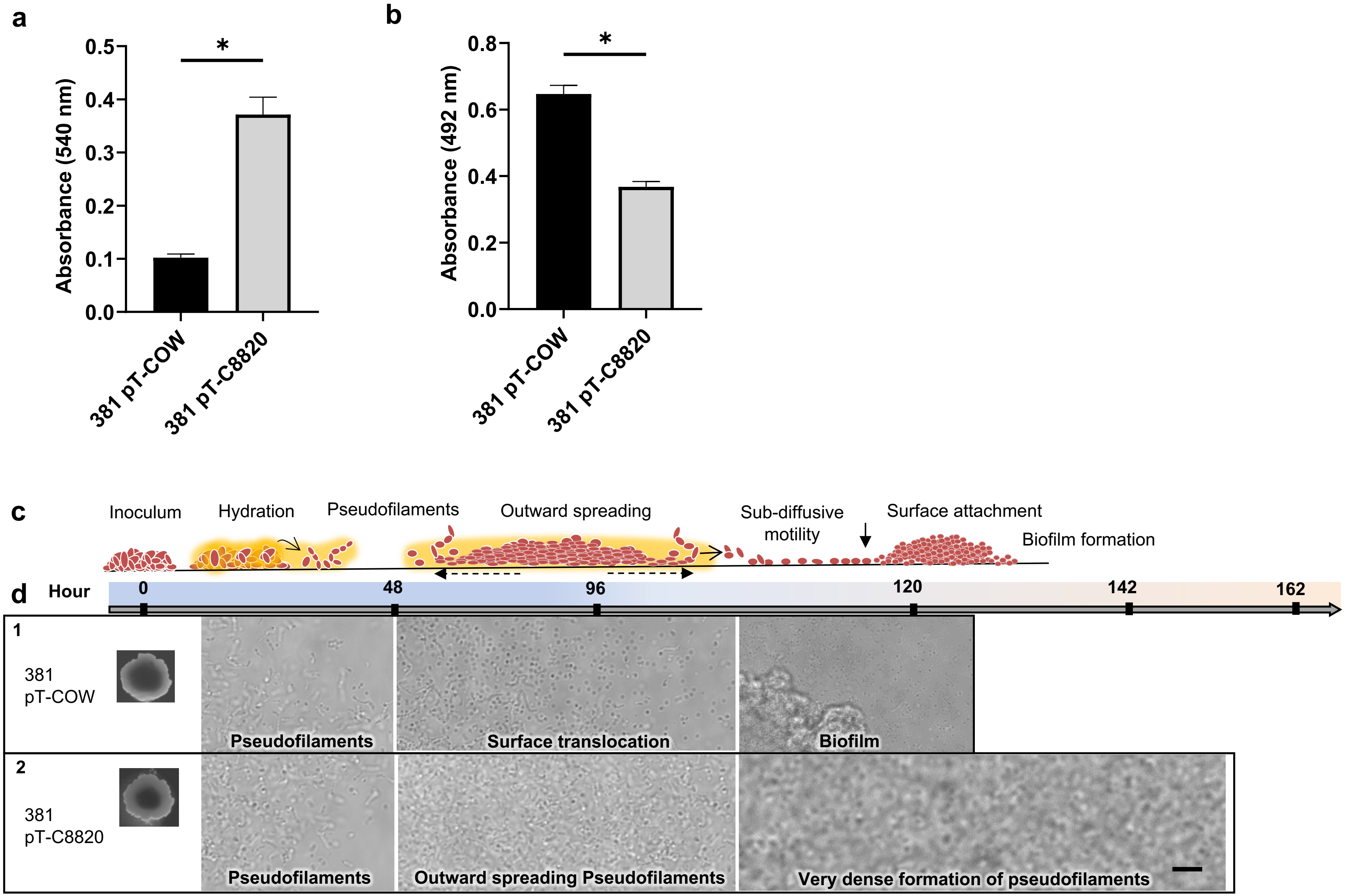


**Supplemental Fig. 5** Testing the effects of overexpressing *ppad* on 381 biofilm formation and surface translocation. (**a**) Overexpression of ppad in strain 381 (381 pT-COW::*ppad*) was confirmed by measuring PPAD enzymatic activity using a colorimetric assay. 381 pT-COW is the empty vector control. (**b**) Overexpression of ppad lowers biofilm formation. (**c**) Schematic illustration representing sequential stages of surface translocation by the reference strain 381 as recorded for more than 160-hour incubation using the chamber slide system. (**d**) Time-lapse microscopic recordings of surface translocation by 381 pT-COW and 381 pT-COW::*ppad* showed that overexpression of *ppad* resulted in the formation of a highly dense population of pseudofilaments in the hydrated area. Scale bar: 10 μm

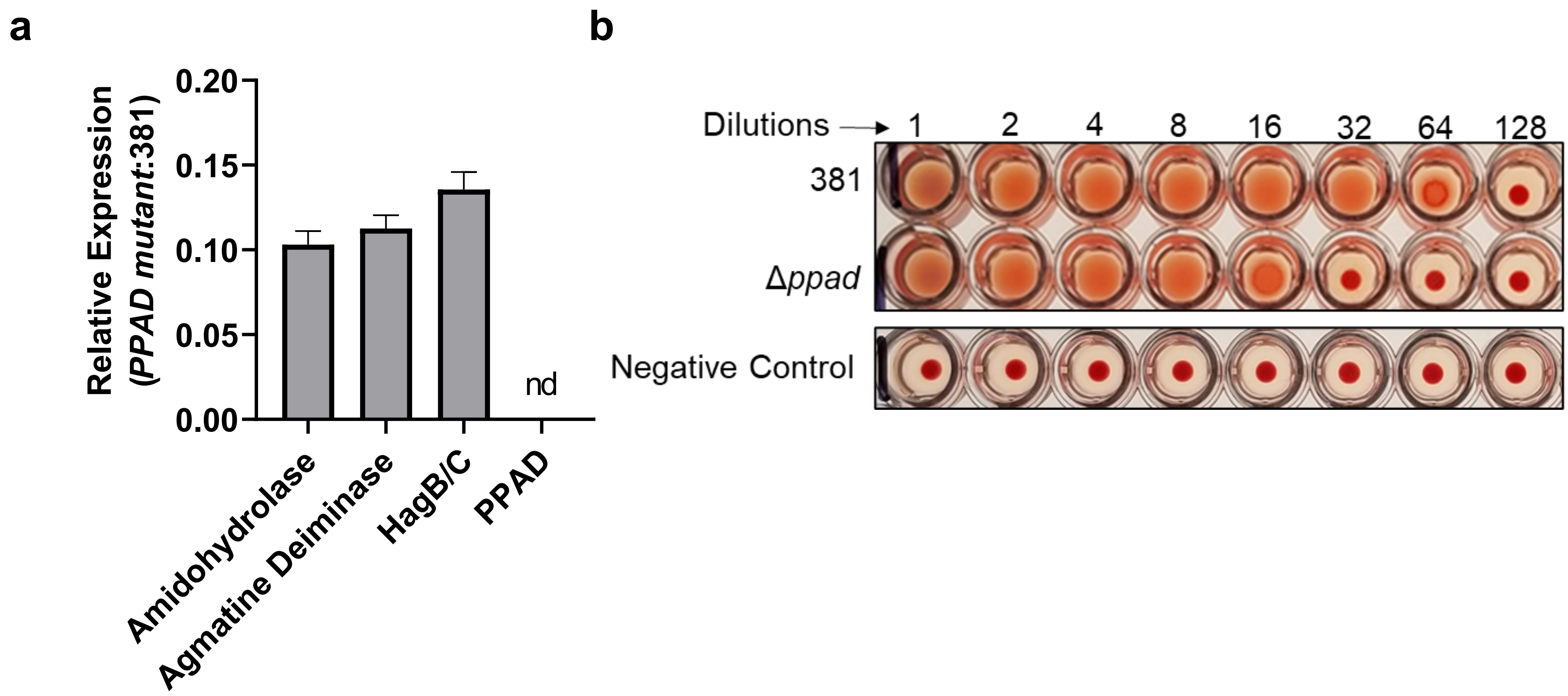


**Supplemental Fig. 6** Experimental verification of select RNA-Seq findings. (**a**) RT-qPCR verification of genes found to be differentially expressed by RNA-Seq confirmed that N-Carbamoylputrescine Amidohydrolase (PGN_0256), Agmatine Deiminase (PGN_0257), HagB (PGN_1904)/HagC (PGN_1906), which are duplicated genes and could not be differentiated, and PPAD (PGN_0898) were all downregulated in 381Δ*ppad*. Data are the average of three replicates; p-values <0.05. Error bars represent the standard error of the mean. (**b**) 381 and 381Δ*ppad* were diluted then mixed with 2% defibrinated sheep erythrocytes and incubated at room temperature for 3 h. 381Δ*ppad* showed lower hemagglutinating activity than parent strain 381.

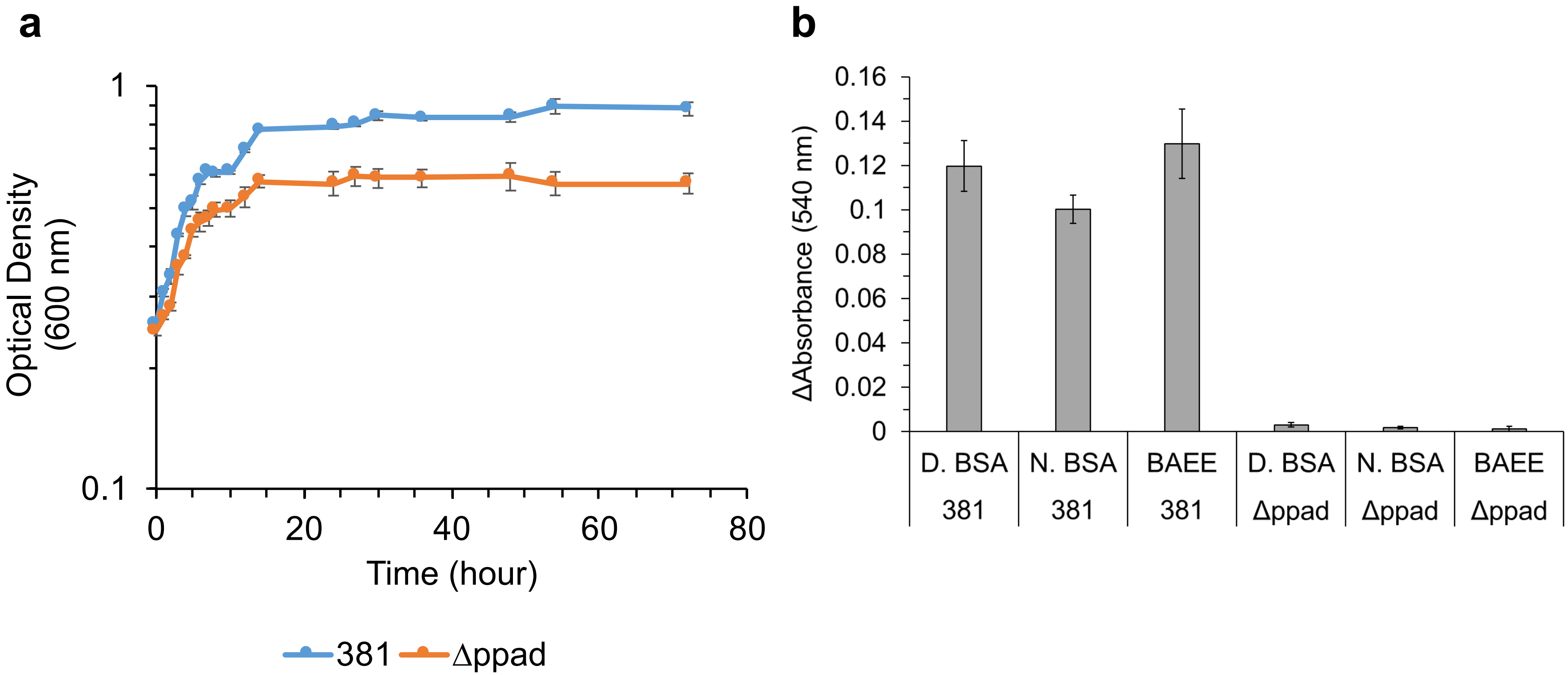


**Supplemental Fig. 7** Ability of 381 and Δ*ppad* to grow in and citrullinate bovine serum albumin (BSA). (**a**) Growth rate of 381 and Δ*ppad* in 1% BSA showed that Δ*ppad* displayed a defect in growth rate compared with the parent strain. (**b**) PPAD enzymatic activity was tested using a colorimetric assay in which denatured BSA (D. BSA), native BSA (N. BSA), or BAEE (control) were used as substrates. 381 citrullinated denatured BSA, native BSA, and BAEE. Δ*ppad* could not citrullinate any substrate. Data represent the average of three replicates. Error bars represent the standard deviation.

**Supplemental Table 1** Differential gene expression in 381Δ*ppad* versus 381 colony biofilms based on RNA-Seq analysis

| Gene number | Predicted molecule/family | LogFC | FDR |
|---|---|---|---|
| predicted RNA | - | -2.6481061 | 0.00026203 |
| PGN_0256 | hydrolase | -2.1592278 | 0.00038042 |
| PGN_0649 | hypothetical protein | -1.8545431 | 0.00058093 |
| PGN_0257 | agmatine deiminase | -1.7498358 | 0.00065416 |
| PGN_0068 | hypothetical protein | -1.7431652 | 0.00040625 |
| PGN_1429 | hypothetical protein | -1.6964042 | 0.00190852 |
| PGN_1906 | hemagglutinin protein HagC | -1.6676265 | 0.00043752 |
| PGN_1904 | hemagglutinin protein HagB | -1.5911948 | 0.0003305 |
| PGN_t0044 | Tyr tRNA | -1.4591436 | 0.0003674 |
| PGN_0694 | 50S ribosomal protein L34 | -1.453808 | 0.00218384 |
| predicted RNA | - | -1.4465263 | 0.00310443 |
| PGN_0258 | preprotein translocase subunit SecG | -1.394757 | 0.0009467 |
| PGN_1872 | 30S ribosomal protein S12 | -1.3751366 | 0.0003305 |
| PGN_0320 | hypothetical protein | -1.3592331 | 0.00412113 |
| PGN_1998 | hypothetical protein | -1.3451009 | 0.00052367 |
| PGN_1874 | 3-phosphoshikimate 1-carboxyvinyltransferase | -1.3048378 | 0.00028838 |
| PGN_1732 | hypothetical protein | -1.2990671 | 0.00061598 |
| PGN_1004 | ABC transporter ATP-binding protein | -1.293211 | 0.0003305 |
| PGN_1659 | ferredoxin | -1.2836596 | 0.0003674 |
| PGN_0640 | 30S ribosomal protein S18 | -1.2388857 | 0.0003674 |

| | | | |
|---|---|---|---|
| PGN_0150 | ATP-dependent RNA helicase | -1.2267062 | 0.00026203 |
| PGN_1364 | peptidyl-prolyl cis-trans isomerase cyclophilin-type | -1.2246059 | 0.00178132 |
| PGN_0872 | DNA-binding protein histone-like family | -1.222751 | 0.0003674 |
| PGN_0498 | succinate dehydrogenase/fumarate reductase iron-sulfur subunit | -1.214574 | 0.00141953 |
| PGN_0503 | biotin carboxyl carrier protein | -1.2017507 | 0.0003305 |
| PGN_1498 | hypothetical protein | -1.1862177 | 0.0003305 |
| PGN_1633 | formiminotransferase-cyclodeaminase | -1.1778902 | 0.00026203 |
| PGN_0698 | nitroreductase | -1.1563353 | 0.0003305 |
| predicted RNA | - | -1.1530753 | 0.00890889 |
| PGN_1973 | phosphoglycerate mutase | -1.1503063 | 0.00038042 |
| PGN_1631 | putative DNA-binding protein histone-like family | -1.1307679 | 0.00038042 |
| PGN_1206 | bifunctional 5,10-methylene-tetrahydrofolate dehydrogenase/ 5,10-methylene-tetrahydrofolate cyclohydrolase | -1.1302895 | 0.00046565 |
| PGN_0433 | phosphoglycerate kinase | -1.1207172 | 0.0003674 |
| PGN_0254 | ParB chromosome partitioning protein | -1.1182703 | 0.00046336 |
| PGN_0714 | pyrazinamidase/nicotinamidase | -1.1108896 | 0.0003674 |
| PGN_1630 | transcription termination factor Rho | -1.1081255 | 0.0003305 |
| PGN_0319 | RNA polymerase sigma-70 factor ECF subfamily | -1.1062575 | 0.00526511 |
| PGN_0255 | ParA chromosome partitioning protein | -1.0982211 | 0.00045575 |
| PGN_1989 | hypothetical protein | -1.0910933 | 0.00038042 |

| | | | |
|---|---|---|---|
| PGN_0114 | Na(+)-translocating NADH-quinone reductase subunit A | -1.0886777 | 0.00038519 |
| PGN_t0032 | Thr tRNA | -1.0845815 | 0.00300271 |
| PGN_0329 | hypothetical protein | -1.0798147 | 0.00038519 |
| PGN_t0040 | Tyr tRNA | -1.0724823 | 0.00261341 |
| PGN_0674 | hypothetical protein | -1.0675723 | 0.00067716 |
| PGN_0138 | hypothetical protein | -1.06597 | 0.00038042 |
| PGN_t0039 | Gly tRNA | -1.0632069 | 0.00416996 |
| PGN_1634 | imidazolonepropionase | -1.0470292 | 0.00222373 |
| PGN_0434 | phosphoenolpyruvate carboxykinase | -1.0390169 | 0.00222373 |
| PGN_0871 | hypothetical protein | -1.0312807 | 0.00053454 |
| PGN_0668 | RNA-binding protein | -1.0207355 | 0.00141477 |
| PGN_1899 | hypothetical protein | -1.0051276 | 0.00403361 |
| PGN_0304 | hypothetical protein | 1.00115956 | 0.00933437 |
| PGN_1766 | hypothetical protein | 1.00231708 | 0.00523557 |
| PGN_0846 | hypothetical protein | 1.00304092 | 0.00526511 |
| PGN_0040 | hypothetical protein | 1.00463084 | 0.0023313 |
| PGN_1624 | hypothetical protein | 1.00525187 | 0.00397173 |
| PGN_0337 | hypothetical protein | 1.01359021 | 0.0055917 |
| PGN_2036 | hypothetical protein | 1.02109288 | 0.00566185 |
| PGN_0050 | hypothetical protein | 1.0260462 | 0.00535462 |
| PGN_1291 | conserved hypothetical protein related to phage | 1.02614012 | 0.00535462 |
| PGN_0074 | hypothetical protein | 1.02716618 | 0.00286151 |

| | | | |
|---|---|---|---|
| PGN_0822 | hypothetical protein | 1.02880994 | 0.00424146 |
| PGN_t0007 | Met tRNA | 1.03431962 | 0.00485043 |
| PGN_1133 | hypothetical protein | 1.03946215 | 0.00890889 |
| PGN_1145 | hypothetical protein | 1.04881036 | 0.00194136 |
| PGN_0479 | hypothetical protein | 1.0535485 | 0.00848419 |
| PGN_1249 | hypothetical protein | 1.05894676 | 0.00756649 |
| PGN_0978 | hypothetical protein | 1.0612413 | 0.00957153 |
| PGN_r0005 | 23S ribosomal RNA | 1.06812875 | 0.00535462 |
| PGN_r0011 | 23S ribosomal RNA | 1.06817006 | 0.00535462 |
| PGN_r0008 | 23S ribosomal RNA | 1.06889164 | 0.00535462 |
| PGN_r0002 | 23S ribosomal RNA | 1.06890623 | 0.00535462 |
| PGN_1560 | hypothetical protein | 1.07024902 | 0.00337349 |
| PGN_1609 | hypothetical protein | 1.14827919 | 0.00300854 |
| PGN_0681 | hypothetical protein | 1.17134298 | 0.00373166 |
| PGN_t0019 | Arg tRNA | 1.20125564 | 0.001616 |

**Supplemental Table 2** Strains used in this study

| **Strain or plasmid (relevant genotype or phenotype)** | **Source or reference** |
|---|---|
| *P. gingivalis* strains | |
| 381 (wild type) | H. Kuramitsu, State University of Buffalo, Buffalo, NY |
| 33277 (wild type) | American Type Culture Collection |
| ΔPGF_8820::Erm ($Em^r$) in strain 381 | (1) |
| ΔPGN_0898::Erm ($Em^r$) in strain 33277 | This study |
| 381 pT-COW (Empty vector control) | This study |
| 381 pT-C8820 (PPAD-overexpressing strain) | This study |
| Plasmids | |
| pT-COW ($Cb^r$, $Tc^r$) | (2) |
| pT-C8820 ($Cb^r$, $Tc^r$) | (1) |

## References


1. Vermilyea DM, Ottenberg GK, Davey ME. 2019. Citrullination mediated by PPAD constrains biofilm formation in *P. gingivalis* strain 381. NPJ Biofilms Microbiomes 5:7.
2. Gardner RG, Russell JB, Wilson DB, Wang GR, Shoemaker NB. 1996. Use of a modified Bacteroides-Prevotella shuttle vector to transfer a reconstructed beta-1,4-D-endoglucanase gene into Bacteroides uniformis and Prevotella ruminicola B(1)4. Appl Environ Microbiol 62:196-202.

**Supplemental Table 3** Primers used in this study

| Primer purpose and name | Sequence (5'-3') |
|---|---|
| PGN_0898 (*ppad*) mutant construction and verification | |
| 1kb upstream PGN_0898 F | GTAGGTCTCTGCGAACCTC |
| 1kb upstream PGN_0898 R | CTTTTTTGTCATTGTTTGATATGTTTTATGATGTTATGAATATG |
| PGN_0898 KO *ermF* F | ATATCAAACAATGACAAAAAAGAAATTGCCC |
| PGN_0898 KO *ermF* R | AAGGCCCCCTACGAAGGATGAAATTTTTCAG |
| 1kb downstream PGN_0898 F | CTTCGTAGGGGGCCTTATTTGAGAATAC |
| 1kb downstream PGN_0898 R | GTAAGGGTTCGCTTAAGATC |
| PGN_0898 KO Cassette Nested F | CCATACACCTGCTCATTGGT |
| PGN_0898 KO Cassette Nested R | TCTGTTTTCCCACCGAAC |
| ΔPGN_0898::*ermF* Seq F | AGACCTTTGCGCAATAAC |
| ΔPGN_0898::*ermF* Seq R | CTTGAGGCGGTAGGAAAC |
| qRT-PCR of PGN_1904 (HagB) | |
| PGN_1904.qPCR_Fw | GAGAAGCAGCCGTGGAGAAG |
| PGN_1904.qPRC_Rv | CCGTTCTCGGCTACGAATGT |
| qRT-PCR of PGN_0256 (Agmatine deiminase) | |
| PGN_0256.qPCR_Fw | GCGATGGTGCCGAAGAAATC |
| PGN_0256.qPCR_Rv | AGCACTCATTCAGCAAGCCA |
| qRT-PCR of PGN_0257 (N-carbamoylputrescine amidohydrolase) | |
| PGN_0257.qPCR_Fw | AAGTCGGCTATCATCGGACG |
| PGN_0257.qPCR_Rv | CTGATAGTCGTTTGCCCCGA |
| qRT-PCR of PGN_0898 (PPAD) | |
| PGN_0898.qPCR_Fw | CCGAACGGATAGCGTACCAA |
| PGN_0898.qPCR_Rv | GCAAATGCAAGCAGACCGAA |

**Supplemental Video Legends**

**Supplemental Video 1.** Time-lapse microscopy recordings of *P. gingivalis* 381 forming outspreading pseudofilaments. Video length: 60 sec; interval: 15 mSec; frame rate: 29 frames/sec.

**Supplemental Video 2.** Time-lapse microscopy recordings very long pseudofilaments formed by *P. gingivalis* 381 Δ*ppad* under surface translocation condition that tend to aggregate. Video length: 60 sec; interval: 15 mSec; frame rate: 29 frames/sec.

**Supplemental Video 3.** Time-lapse microscopy recording shows that *P. gingivalis* 381 Δ*ppad* form scattered biofilms in the hydrated area much earlier than the parental strain.

**Supplemental Video 4.** Time-lapse microscopy recordings of sub-diffusive cell-driven motility by *P. gingivalis* about 120 hours into surface translocation course. Video length: 60 sec; interval: 15 mSec; frame rate: 29 frames/sec.

**Supplemental Video 5.** Time-lapse microscopy recordings of sub-diffusive cell-driven motility by *P. gingivalis*381 Δ*ppad* about 160 hours into surface translocation course. Video length: 60 sec; interval: 15 mSec; frame rate: 29 frames/sec.
